# Phage-Mediated Digestive Decolonization in a Gut-On-A-Chip Model: A Tale of Gut-Specific Bacterial Prosperity

**DOI:** 10.3390/v16071047

**Published:** 2024-06-28

**Authors:** Brieuc Van Nieuwenhuyse, Maya Merabishvili, Nathalie Goeders, Kevin Vanneste, Bert Bogaerts, Mathieu de Jode, Joachim Ravau, Jeroen Wagemans, Leïla Belkhir, Dimitri Van der Linden

**Affiliations:** 1 Institute of Experimental and Clinical Research, Pediatric Department (IREC/PEDI), Université Catholique de Louvain—UCLouvain, 1200 Brussels, Belgium; 2 Laboratory for Molecular and Cellular Technology, Queen Astrid Military Hospital, 1120 Brussels, Belgium; 3 Transversal Activities in Applied Genomics, Sciensano, Juliette Wytsmanstraat 14, 1050 Brussels, Belgiumbert.bogaerts@sciensano.be (B.B.); 4 Bacterial Diseases, Sciensano, Juliette Wytsmanstraat 14, 1050 Brussels, Belgium; 5 Laboratory of Gene Technology, KU Leuven, 3000 Leuven, Belgium; jeroen.wagemans@kuleuven.be; 6 Division of Internal Medicine and Infectious Disease, Cliniques Universitaires Saint-Luc, Université Catholique de Louvain—UCLouvain, 1200 Brussels, Belgium; 7 Louvain Centre for Toxicology and Applied Pharmacology, Institute of Experimental and Clinical Research (IREC/LTAP), Université Catholique de Louvain—UCLouvain, 1200 Brussels, Belgium; 8 Pediatric Infectious Diseases, General Pediatrics Department, Cliniques Universitaires Saint-Luc, Université Catholique de Louvain—UCLouvain, 1200 Brussels, Belgium

**Keywords:** bacteriophage, *E. coli*, *P. aeruginosa*, Gut-On-A-Chip, digestive decolonization, mucus

## Abstract

Infections due to antimicrobial-resistant bacteria have become a major threat to global health. Some patients may carry resistant bacteria in their gut microbiota. Specific risk factors may trigger the conversion of these carriages into infections in hospitalized patients. Preventively eradicating these carriages has been postulated as a promising preventive intervention. However, previous attempts at such eradication using oral antibiotics or probiotics have led to discouraging results. Phage therapy, the therapeutic use of bacteriophage viruses, might represent a worthy alternative in this context. Taking inspiration from this clinical challenge, we built Gut-On-A-Chip (GOAC) models, which are tridimensional cell culture models mimicking a simplified gut section. These were used to better understand bacterial dynamics under phage pressure using two relevant species: *Pseudomonas aeruginosa* and *Escherichia coli*. Model mucus secretion was documented by ELISA assays. Bacterial dynamics assays were performed in GOAC triplicates monitored for 72 h under numerous conditions, such as pre-, per-, or post-bacterial timing of phage introduction, punctual versus continuous phage administration, and phage expression of mucus-binding properties. The potential genomic basis of bacterial phage resistance acquired in the model was investigated by variant sequencing. The bacterial “escape growth” rates under phage pressure were compared to static in vitro conditions. Our results suggest that there is specific bacterial prosperity in this model compared to other in vitro conditions. In *E. coli* assays, the introduction of a phage harboring unique mucus-binding properties could not shift this balance of power, contradicting previous findings in an in vivo mouse model and highlighting the key differences between these models. Genomic modifications were correlated with bacterial phage resistance acquisition in some but not all instances, suggesting that alternate ways are needed to evade phage predation, which warrants further investigation.

## 1. Introduction

Infections due to drug-resistant organisms, including antimicrobial-resistant bacteria, have become a major global health threat. These are estimated to be responsible for around 1.2 million yearly deaths, while recent predictions have warned that this could increase to up to 10 million by 2050, especially affecting low- and middle-income countries, if no significant action is taken to curb this trend [1,2]. In the hospital setting, specific subsets of patients may be subjected to various screening programs to detect asymptomatic carriages of certain multi- (and, by extension, extensively- and pan-) drug-resistant (MDR/XDR/PDR) bacteria to optimize the prevention and control of hospital-acquired infections. Among them, rectal swab admission screening is considered a cost-effective and efficient method to detect asymptomatic digestive carriage of certain MDR bacteria of interest, including Gram-negative bacilli, such as extended-spectrum beta-lactamase-producing or carbapenemase-producing Enterobacterales (ESBL-PE/CPE), or other MDR Gram-negative bacilli, such as MDR-*Pseudomonas aeruginosa* [3,4]. These screening results allow for preventive measures, such as patient isolation, to be carried out, which can limit the nosocomial spread of these bacteria [5].

Besides their role in limiting nosocomial spread, rectal swab screening results can also benefit the screened patients themselves by refining their infectious risk profiles. Indeed, the asymptomatic digestive carriage of the aforementioned MDR bacteria puts carriers at a significantly increased risk of later developing an infection caused by the carried bacteria, with the magnitude of this risk varying according to other risk factors such as intensive care unit stay, major abdominal surgery, and hematological conditions including immune suppression [6,7,8]. Hence, the morbidity, mortality, and costs associated with these infections foster the need for preventive suppression strategies for these carriages. However, previous suppression strategies such as oral antibiotic decolonization, oral probiotic regimens, as well as limited experience with fecal microbiota transplantation have yielded disappointing results, reaching temporary suppression at best rather than durable eradication [8]. In addition, oral large-spectrum antibiotic regimens also present potentially serious side effects regarding gut microbiota modification and antimicrobial resistance induction, and fecal microbiota transplantation, although generally safe, has led to serious and sometimes fatal adverse events on rare occasions [8,9]. This discouraging situation can thus be considered as an unmet medical need for alternative or complementary suppression approaches, which warrant further research. Phage therapy (PT) has been mentioned among them [8].

PT is the use of selected viruses infecting bacteria (called bacteriophages, or phages for short) as bactericidal agents in replacement or in combination with antibiotics. Phages are viruses that specifically prey on bacteria and are naturally found in various environmental samples. Recently, the use of PT in the treatment of various bacterial infections that are often difficult to treat has been rekindled in the Western world, along with the study of its safety and efficacy [10]. Both case-based and trial-based studies in the literature unanimously report the general safety of PT regardless of the administration route, though reversible and non-clinically significant cases of transaminitis have been reported on rare occasions [10,11,12]. This favorable safety profile might be partly attributed to phages’ very narrow bactericidal spectrum, conditioned by complex bacterial surface receptor recognition. This makes phages ideal candidates for species-specific bactericidal therapy, minimizing collateral damage on commensal microbiota. PT efficacy is still mostly, though not exclusively, appreciated through case-based studies in the literature, which have recently reported impressive salvage therapies in situations that reached therapeutic dead-ends [13,14].

PT can be delivered in a number of administration routes to target various body compartments, such as topical administration on surface wounds, in situ instillations in abscess cavities, pulmonary nebulization, or intravenous infusion. However, among them, the oral administration of phages to target bacteria in the digestive tract is notoriously challenging and has occasionally yielded disappointing results in both animal and human applications [15,16]. Interestingly, this contrasts with the well-documented massive presence and impact of the resident gut commensal phage community, whose complex role in shaping the bacterial compartment of the gut microbiota in both health and disease is increasingly investigated [17,18,19]. Green and colleagues recently illustrated that the digestive tract’s inhospitable nature towards oral phage therapy was at least partly caused by one key inhibitory compound: mucin [16]. Moreover, they showed that this inhibitory effect could be surpassed by the administration of a phage featuring unique mucus-binding properties (MBPs), resulting in significantly increased lytic power against a gut-colonizing *Escherichia coli* strain (subtype ST131) in both in vitro and mice models compared to another anti-*E. coli* phage devoid of MBPs.

In this paper, we translate the clinical challenge of phage-mediated targeted digestive decolonization into a preliminary yet complex in vitro pre-clinical model: the Gut-On-A-Chip (GOAC). GOACs are part of a larger family of microfluidic tridimensional tubular cell culture models, human organs-on-chips, whose development has been directed and published by Donald Ingber and colleagues since 2010 [20]. Since then, they have published thorough microfabrication and handling protocols that allowed others to reproduce these models and adapt them to their specific research needs and organs of interest [21]. Some have since brought a proof of concept that GOACs can be fine-tuned to high degrees of complexity, aiming for maximum physiological relevance, including the ability to sustain the growth of a complex pseudo-gut microbiota [22]. Others, including phage researchers, have used simpler GOAC designs to model a smaller subset of chosen key characteristics without relying on complex materials and scarce technology [23,24]. Here, taking inspiration from the latter, we use custom GOACs to investigate the quantitative and qualitative aspects of bacterial dynamics under phage pressure in simplified gut-like conditions, including the impact of phage MBPs, focusing on two bacterial species of interest: *P. aeruginosa* and *E. coli*.

## 2. Materials and Methods

### 2.1. GOAC Microfabrication—Inorganic Scaffold Assembly

**Figure 1:**
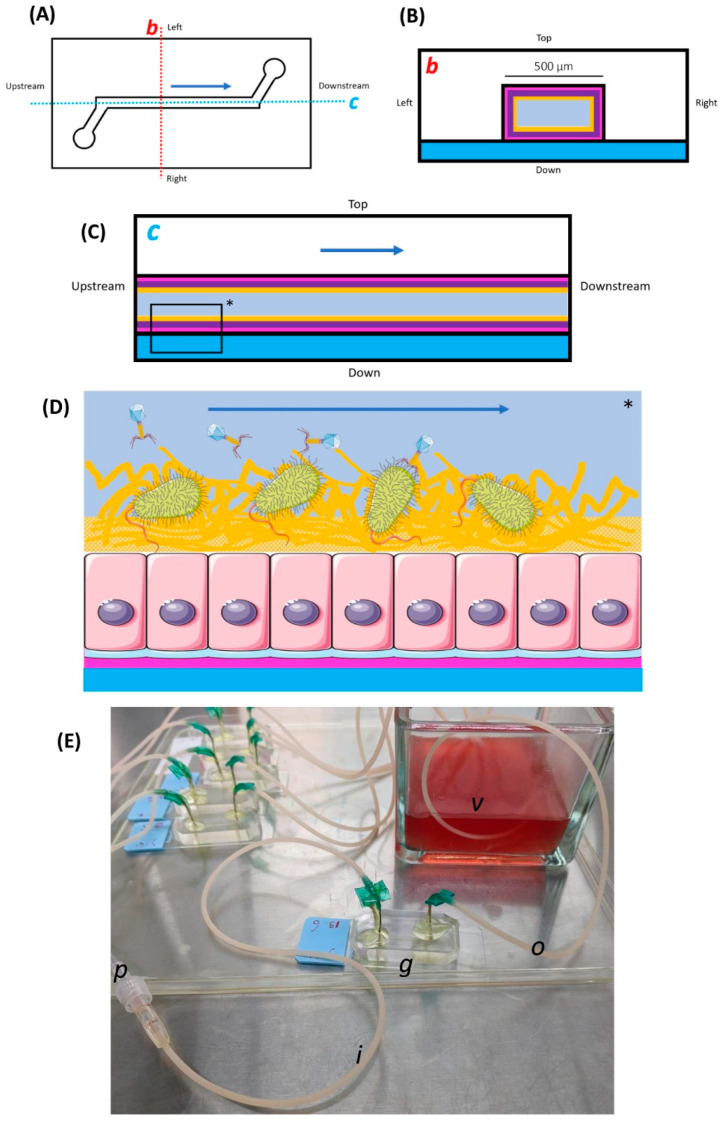
Schematic and photographic views of the Gut-On-A-Chip (GOAC). (**A**) A schematic upper view of the GOAC pattern. The lower left and upper right circles represent the inlet and outlet pores, respectively, between which the GOAC channel is carved. The dark blue arrow represents the direction of the microfluidic flow of the culture medium in the GOAC. The dotted line axes b (red) and c (blue) are used for further schematic representations. (**B**) The transversal section in the GOAC is shown along axis b. The floor of the GOAC is a glass slide (blue) on which a carved polydimethylsiloxane (PDMS) chip (white) is sealed, between which the GOAC channel itself is delimitated. The several concentric layers of the GOAC’s “gut wall” are represented, from outer to inner: the extra-cellular matrix (pink), epithelium (violet), parietal mucus layer (yellow), and the central flow of the culture medium (grey). (**C**) The longitudinal section in the GOAC is shown along axis c. The elements are similar to those of the transversal section. (**D**) A schematic zoom in a segment (*) of the longitudinal section. In this work, the GOAC model is designed to recapitulate the scenario of phage therapy targeting bacteria in the digestive micro-environment, which includes a parietal mucus layer. (**E**) A picture of a GOAC. The GOAC itself (g) is connected to the inlet (i) and outlet (o) winged catheters, respectively, introducing and evacuating all reagents to and from the GOAC. The winged catheters’ needles are manually curved to be punched in inlet and outlet pores, forming a 90° angle with the PDMS chip surface. At the point where the needles are punched into the PDMS chip, epoxy glue is applied to ensure impermeability. The culture medium is infused by a microfluidic pump connected to the inlet catheter by a connecting catheter (p). Egressing medium coming out of the GOAC through the outlet catheter is evacuated in a glass collecting vessel (v) filled with a base level of 70% *v*/*v* isopropyl alcohol (IPA).

Our GOAC microfabrication protocol results from a mix of previously described elements from the aforementioned works of Huh et al., 2013 [21], Jalili-Firoozinezhad et al. [22], 2019, and Chin and Barr, 2019 [23], as well as occasional personal adaptation through trial and error. We chose to work with single-channel GOAC models similar to the one described by Chin and Barr, 2019, as opposed to the double-channel GOAC models described by Huh et al., 2013 [21] and Jalili-Firoozinezhad et al., 2019 [22]. A schematic view of the GOAC models used in our work is displayed in Figure 1.

The general scaffold of the single-channel GOAC relies on the bonding of a polydimethylsiloxane (PDMS) chip onto a glass slide, between which the GOAC’s internal channel volume, carved into the PDMS chip, is delimitated. This requires the creation of a custom mold in which PDMS will be poured and shaped into the desired pattern. Our mold was built using a plastic 3D printer (UltiMaker 3, UltiMaker B.V., Utrecht, the Netherlands) and associated software (UltiMaker Cura, v4.11, UltiMaker B.V., the Netherlands) after modeling the desired pattern file through an online 3D-printing-modelization platform (Tinkercad, Autodesk Inc., San Rafael, CA, USA). PDMS and its curing agent (Sylgard-184 Silicone Elastomer Kit, Dow Inc., Midland, MI, USA) were then poured in a disposable plastic weighing dish at a recommended 10:1 mass ratio and thoroughly mixed by hand using a stainless steel lab spatula for 5 min. The mixture was then left to rest at room temperature for about 30 min before residual bubbles were removed with the tip of a sterile needle.

The PDMS mixture was then manually poured in the mold at the most gentle and constant pace possible until the mold was full. The full mold was then left to rest and cure at room temperature for at least 36 h before the fully solidified PDMS chip was carved out of the mold with the help of a surgical blade or a lab spatula. Between each use, the mold was thoroughly washed by being brushed under tap water followed by 70% *v*/*v* isopropyl alcohol (IPA) (20842.330, VWR international, Radnor, PA, USA) to ensure the full clearance of PDMS residue, and then it was left to evaporate under a fume hood until completely dry before it was used again. Carved PDMS chips were then further cured in a dry incubator at 60 °C for at least one hour with the channel side up. Their four corners were trimmed using a surgical blade to give the chips an octagonal shape while ensuring the cuts did not become close to the molded channel pattern. Finally, the chips were bathed in 70% *v*/*v* IPA and left to fully dry before being stored in any clean container.

Plasma bonding of the PDMS chips to glass slides (Superfrost Plus, Thermo Fischer Scientific (Menzel-Gläser),Waltham, MA, USA) was then performed. Right before the PDMS chip and the glass slide underwent plasma treatment, the glass slide was gently wiped clean with 70% *v*/*v* IPA and left to dry fully while visually ensuring the PDMS chip and wiped slide were devoid of any residue or dust particle. The PDMS chip (channel side up) and the glass slide were placed in the barrel of the plasma cleaner (TePla 300E, PVA TePla, Wettenberg, Germany). The venting valve of the plasma cleaner was opened on a high throughput so as to minimize post-plasma venting delay. O_2_ plasma treatment was initiated at 70 watts for 45 s. As soon as venting was complete and the plasma cleaner’s door could be opened, the glass slide was swiftly taken out while leaving the plasma-treated area untouched with the intention of bonding to the PDMS chip. The PDMS chip was then also taken out without touching its plasma-treated face (channel face), and then the respective plasma-treated faces of the PDMS chip and the glass slide were manually pressed against each other. The plasma-induced surface activation effects wore off quickly; the delay between door opening and face-to-face application should not exceed ten seconds. Both parts were pressed firmly together using fingers without moving for at least 30 s. Then, to strengthen the plasma bonding, annealing of the assembled chip was performed by placing it in an oven at 80 °C for at least two hours. The chip was then taken out of the oven and stored in a clean container for later use. These chips formed the inorganic scaffold of the future GOAC models.

### 2.2. GOAC Microfabrication—Cell Culture

This part of the GOAC microfabrication process can be divided into three successive steps: (i) inlet and outlet catheter connection, (ii) extra-cellular matrix (ECM) coating, and (iii) cell seeding.

Inlet and outlet catheters were needed to, respectively introduce and evacuate all reagents, cells, bacteria, and phages from the GOAC, including the continuous unidirectional flow of culture medium infused in the GOAC to sustain cell growth (see below); they formed an upstream and downstream tubular continuity with the actual GOAC channel inside the chip. Winged 21G catheters (21BLK03, Terumo, Tokyo, Japan) were used after cutting their wings short with a surgical blade and manually bending their needle to give it a smooth ~90° curvature. The curved needles were then manually punched inside the PDMS chip’s inlet and outlet pores, forming a ~90° angle with the chip’s upper surface. Punching needles sufficiently deep and accurately inside the channel pores is critical to ensure future top-to-bottom permeability in the system. To ensure the needle–PDMS junction was durably impermeable, once the needles were punched in place, the needle–PDMS junctions at the surface of the chip were covered in two-component epoxy glue (Super Mix Universal, Power Epoxy, Pattex, Henkel, Düsseldorf, Germany), which was then left to solidify at room temperature for at least one hour. Chips were then placed under UV light overnight for sterilization.

The GOAC’s inner walls were then coated with ECM solution. The end of the GOAC’s outlet catheter was placed in a collecting vessel that collected egressing fluid from the GOAC from then on. When working on a series of several GOACs at the same time, GOACs seeded with different cells, bacteria, or phages should always have their outlet catheters placed in separate collecting vessels to mitigate the cross-contamination risk. A base level of 70% *v*/*v* IPA was poured in this collecting vessel for further instant sterilization of the egressing fluid. To prepare 10 mL of ECM solution under the laminar flow, the following refrigerated (4 °C) reagents were poured in a 50 mL sterile tube (Cellstar, Greiner bio-one, Frickenhausen, Germany) put in ice: 9800 µL of serum-free DMEM (41965-039, Gibco, Thermo Fischer Scientific, Waltham, MA, USA), 180 µL of rat tail type I collagen (354236, Corning, NY, USA), and 20 µL of Matrigel (354234, Corning, USA). This ECM solution was then gently mixed by manually shaking the closed tube before putting it back in ice. This refrigerated ECM solution must be used immediately. A 3 mL sterile plastic syringe was loaded with 1.5 mL of ECM solution. Loading the syringe very slowly reduces bubble formation. The filled syringe was then connected to the GOAC’s inlet catheter before the ECM solution was gently infused through the GOAC until egressing ECM was visible at the end of the outlet catheter. A visual check ensures no visible bubble is present in the ECM-filled GOAC since these spots would then remain uncoated. The ECM-filled GOAC was placed in a humidified cell culture incubator (37 °C; 5% CO_2_) overnight with the ECM syringe still connected to the inlet catheter.

The next day, the ECM excess fluid was gently flushed out of the GOAC by slowly instilling, with a syringe connected to the inlet catheter, 1.5 mL of the following culture medium that was used from then on (“FULL medium”): DMEM supplemented with 20% fetal bovine serum (10270-106, Gibco, Thermo Fischer Scientific, USA), 1% penicillin-streptomycin (15140-122, Gibco, Thermo Fischer Scientific, USA), and 1% non-essential amino acids (11140-050, Gibco, Thermo Fischer Scientific, USA). The GOAC was then ready for cell seeding. In this work, according to tested conditions (see the Section 3), GOACs were either seeded with 100% Caco-2 cells (HTB-37, American Type Culture Collection-ATCC, USA) or with 50-50% Caco-2/LS 174T cells (CL-188, ATCC, Manassas, VA, USA). Once, a triplicate of GOACs was seeded with 100% LS 174T cells for the purpose of comparative ELISA validation only (see below), but were not used for infection assays. Caco-2 cells and LS 174T cells required for GOAC seeding were retrieved by trypsinization, centrifugation, and the resuspension of 90% confluent T175 culture flasks previously cultured with FULL medium. With the help of trypan blue cell counting calibration, a 1 mL sterile syringe was loaded with 1 mL of cell suspension at a total cell seeding density of about 1.5 × 10^7^ cells/mL. This syringe was then connected to the inlet catheter and instilled in full in the GOAC; to ensure cell delivery in the GOAC was sufficient, this was performed with the GOAC’s channel under a microscope. The GOAC was then put back statically in the incubator to ensure cell adhesion to the GOAC’s walls, with the 1 mL syringe still connected, for four hours. The GOAC was then instilled with 1 mL of FULL medium to flush out unattached cells from its lumen. The GOAC was then put back statically in the incubator overnight, with the flushing syringe still attached, to ensure cell growth to epithelial confluence.

The next day, the GOAC was ready to be put under microfluidic infusion. Microfluidic infusion was ensured using a 12-channel microfluidic pump (Braintree Scientific, Braintree, MA, USA) in which 3 mL sterile syringes containing FULL medium were loaded. The GOAC was taken out of the incubator and its inlet catheter was connected to the 3 mL syringe loaded in the pump with the help of a long connecting catheter (PN3120, CAIR L.G.L., Lissieu, France) also pre-filled with FULL medium. The connecting catheter’s length ensures the GOAC can still be moved in and out of the incubator while the pump stays outside the incubator. The GOAC was placed back in the incubator with the connecting catheter passing through the incubator door’s rubber joint. The pump was started with an infusion rate set to 60 µL/h (1 µL/min) for 72 h, to sustain epithelial growth and parietal mucus secretion under microfluidic conditions. At least 12 h before inoculating the GOAC (see the next section), the medium has to be switched from FULL to antibiotic-free medium (“NAB medium”), which has the same composition as the FULL medium except for the absence of 1% penicillin-streptomycin. To connect the GOAC to the NAB medium for more than 12 h, the connecting catheter was first disconnected from the GOAC’s inlet catheter and flushed of its inner FULL medium with new NAB medium using a 3 mL syringe loaded with NAB medium. This 3 mL syringe was then fully refilled with NAB medium, connected again to the connecting catheter, and loaded in the pump. The downstream end of the connecting catheter could then be reconnected to the GOAC’s inlet catheter. This ensured the NAB medium was delivered right from the inlet catheter once the pump was re-started. After more than 12 h of NAB medium infusion, the pump rate was doubled to 120 µL/h (2 µL/min).

### 2.3. GOAC Microfabrication—Bacteria and Phage Introduction and Monitoring

After at least 12 h of NAB medium infusion, bacteria and phages can be introduced in the GOAC. In this work, GOACs were either inoculated with the *P. aeruginosa* strain CN573 and the corresponding anti-*P. aeruginosa* phage named PNM (both sourced from the Laboratory of Molecular and Cellular Technology, Queen Astrid Military Hospital, Brussels, Belgium) or with *E. coli* subtype ST131 and corresponding anti-*E. coli* phages named HP3 and ES17 (all three sourced from the Baylor College of Medicine, Houston, TX, USA). CN573 and ST131’s antibiotic susceptibility testing was performed by standard automated disk-diffusion read by Adagio (Bio-Rad, Hercules, CA, USA) and is included as Supplementary Material (Table S1).

According to the desired test condition (see the Section 3), GOACs were either infected with 1 × 10^7^ CFU (colony forming unit)/mL or 1 × 10^5^ CFU/mL bacterial solutions. Prior to introduction in the GOAC, both *P. aeruginosa* and *E. coli* were subcultured in a 37 °C incubator overnight on self-made LB agar slants. The slant was then washed by adding 5 mL of NAB medium and vortexing the slant tube at a high speed for 10 s, providing an initial bacterial solution that was then systematically titered by serial dilutions in phosphate-buffered saline (Dulbecco’s PBS, PBS-1A, Capricorn Scientific, Ebdorsfergrund, Germany) and plating on 5% sheep blood Columbia agar plates (254071, Becton Dickinson, Franklin Lakes, NJ, USA). The agar plates were incubated overnight, and the titer of the original bacterial suspension was determined post hoc by CFU counting. By experience, this initial solution’s titer was reliably comprised in the 1–2 × 10^9^ CFU/mL interval for both bacterial species, and thus diluted 100 and 10,000 times to reach the aforementioned desired titers of ~1 × 10^7^ and ~1 × 10^5^ CFU/mL, respectively. Variation among the exact infective titers (determined post hoc the next day by plate CFU count, as explained) was neutralized by standardizing each initial titer on a conventional zero and expressing subsequent variation in the GOAC’s infection on a log10 basis (see the Section 2.4).

Phages are similarly titered to establish their PFU (plaque forming unit)/mL titer using standard double agar overlay (DAO) method [25]. Knowing the respective CFU/mL and PFU/mL titers of the instilled bacterial and phage solutions, the multiplicity of infection (MOI, the ratio of phages per bacteria in presence) can be determined when applicable: in this work, the tested MOI is either 1 or 10. In preventive assays in which bacteria-free GOACs are pre-treated with phages, the inner GOAC volume is filled with phage solution at 10^8^ PFU/mL and then statically incubated at 37 °C/5% CO_2_ before being reconnected to the pump and washed with NAB medium either perfused at 1 mL/h for 90 min (“hard wash” protocol) or at 120 µL/h for 12 h (“soft wash” protocol).

According to the tested conditions, the aforementioned bacterial loads and phage loads were introduced in the GOAC, either by direct manual syringe instillation before immediately reconnecting the GOAC to the pumping system or by pump instillation starting from the pump’s syringe all the way to the connecting catheter and the GOAC. “Day 0” always marks the time of initial bacterial introduction to the GOAC. The evolution of the model’s bacterial titer was then assessed every 24 h for 72 h. To achieve this monitoring, 10 µL of egressing fluid were pipetted out of the outlet catheter terminal cupule by inserting a fine pipette tip inside the lumen of the end of the outlet catheter. Through serial dilutions of this 10 µL output, daily in-model CFU/mL counts were established using the same spread plate and colony count technique as that mentioned above.

### 2.4. GOAC Statistics

GOAC statistics are computed using SPSS (IBM Corp. Released 2020. IBM SPSS Statistics for Windows, Version 27.0. Armonk, NY, USA: IBM Corp). Between and within comparisons of the GOACs’ bacterial counts expressed in log10 basis over the days are assessed through repeated measures ANOVA (analysis of variance) tests, with the “within” variable being time (days) and the “between” variable being the different tested conditions. Greenhouse–Geisser correction is automatically applied if Mauchly’s sphericity test’s null hypothesis is not met. ANOVA’s main effects comparison is obtained after Bonferroni correction. Post hoc multiple paired comparisons are generated after Bonferroni correction. All error bars on graphs represent 95% confidence intervals (CI95). Significance is always based on α = 0.05. All tested conditions are biologically independent triplicates (i.e., independent GOACs; n = 3 generally tested in at least two different runs to mitigate batch effect), with each of these conditions’ CFU/mL output being subjected to technological duplicate (mean count based on spread plating on two agar plates). These evolutions of log10 basis counts are centered on the aforementioned control count of the introduced CFU/mL at day 0, which is subtracted to express all further values in comparison to a conventional zero; the graphs thus represent the loss, gain, or status quo of bacterial titer in the model compared to the bacterial titer initially instilled in the model.

### 2.5. GOAC Visuals

To illustrate the tissue architecture of the GOACs, direct in-GOAC coloration was performed using Alcian Blue, staining acidic mucins. We performed a classic Alcian Blue staining protocol, which is usually performed on histological sections, but by infusing and flushing the reagents directly in the GOAC channel instead, via the aforementioned inlet catheter. We used Alcian Blue 8GX (361180-0005, RAL Diagnostics, Martillac, France). The GOAC on which staining was performed was first gently flushed manually with phosphate-buffered saline (Dulbecco’s PBS, Capricorn Scientific GmbH, Ebdorsfergrund, Germany) and then infused with 4% *v*/*v* paraformaldehyde (Q Path, VWR chemicals, VWR international, Radnor, PA, USA) and left to statically fixate for 10 min at room temperature. GOAC was then flushed with PBS again before mordanting was performed, infusing 3% *v*/*v* acetic acid (based on Acetic Anhydride, ref 33214-M, Sigma-Aldrich, Darmstadt, Germany) before 3 min of static incubation was carried out at room temperature. Alcian Blue (1% *v*/*v*) was then infused in the GOAC and left to incubate in a dry incubator at 37 °C for 30 min before being finally flushed manually with distilled water. Stained GOACs were then directly observed through light microscopy. Their stained inlet catheters, found to often contain similarly cellularized segments, were used to obtain 3 µm thick histological sections after a classic tissue preparation and paraffin embedding protocol.

### 2.6. OmniLog Assays—Bacterial Growth Kinetics

To compare GOACs’ bacterial dynamics to other in vitro conditions over an identical timeframe of 72 h, bacterial growth curves were established using an OmniLog incubator system (Biolog, Hayward, CA, USA). Experiments were carried out in 96-well plates in a final volume of 200 μL of LB medium supplemented with 100 times diluted tetrazolium dye mix A according to the manufacturer’s instructions. Control assays were also performed using 20% FBS-supplemented LB instead, mixed with the same dye. Bacteria were added at a concentration of 10^5^ CFU/well, calculated based on optical density (OD, at 600 nm) measurements (with an OD of 0.5 corresponding to 4 × 10^8^ CFU/mL, on average), which were validated using a classical plate culture method. MOI tested in OmniLog assays was 1 for *P. aeruginosa* assays and either 1 or 10 for *E. coli* assays.

OmniLog data were analyzed using OmniLog data Analysis Software (v1.7). OmniLog growth curves based on these results represent bacterial proliferation in various conditions and are presented through relative units of cellular respiration over time. Error bars in these graphs represent +/−1 standard deviation of the mean. Positive and negative control conditions were replicated in 16 wells in each assay. In LB-only assays, *P. aeruginosa* with PNM at MOI = 1 was replicated in 32 wells. *E. coli* assays with either phage ES17 or HP3 were replicated in 24 wells at MOI = 1 and in 8 wells at MOI = 10. In control assays using serum-supplemented LB, all bacteria–MOI combinations were replicated in 24 wells. Comparison of bacterial “escape” growth rate between OmniLog assays and GOAC assays was performed using Fischer’s exact test.

### 2.7. Bacteria and Phages: Description and Variant Typing

Two bacterial species were used in this work: *P. aeruginosa* (strain CN573) and *E. coli* (extraintestinal pathogenic *E. coli*–ExPEC-subtype ST131 isolate JJ1901). *P. aeruginosa* was subjected to only one phage in this work, lytic phage PNM of the Autographiviridae family (GenBank: OP292288); both *P. aeruginosa* CN573 and phage PNM samples were obtained from the Laboratory of Molecular and Cellular Technology (LabMCT, Queen Astrid Military Hospital, Brussels, Belgium). *E. coli* was subjected to two lytic phages: phage ES17 and phage HP3. Phage HP3 (GenBank: GCA_002619885.1) is a lytic *Escherichia*-phage member of the Straboviridae family possessing a myovirus morphology and depending at least partly on *E. coli* lipopolysaccharide (LPS) as a bacterial surface receptor [26]. The more recently described phage ES17 (GenBank: GCA_009744855.2), whose bacterial receptor is not yet characterized, is another lytic *Escherichia*-phage member of the Gordonclarkvirinae subfamily and Kuravirus genus and displays an atypical podovirus-like morphology featuring an elongated capsid and short tail fibers [16,27]. In addition, phage ES17 has recently been described for its MBPs compared to their absence in phage HP3 [16]. Both of these phages, along with the *E. coli* ST131 JJ1901 strain, were obtained from the Baylor College of Medicine (Houston, TX, USA).

Some experiments in this work have generated variants of the aforementioned bacterial strains, initially identified on a visual basis by examining colonies on aforementioned 5% sheep blood Columbia agar plates. Some of these variants have been screened for acquired phage resistance by re-performing aforementioned DAO assays to establish relative efficiency of plating (EOP), the relative ability of a given phage load to achieve lysis plaques on a bacterial lawn of a given bacterial isolate compared to the phage’s reference bacterial host, expressed as a ratio theoretically comprised between 0 and 1. All of the analyzed variants were confirmed to belong to their alleged bacterial species by Matrix-Assisted Laser Desorption Ionization–Time Of Flight (MALDI-TOF). Some variants of *P. aeruginosa* and *E. coli* were also sequenced to try to investigate potential genomic basis for their phenotypic modifications; for reasons of chronological and technical constraints as well as respective expertise, *P. aeruginosa* and *E. coli* sequencing were performed by different teams using different protocols. *P. aeruginosa* sequencing was performed as follows: Total genomic DNA was extracted using the MagCore Genomic DNA Bacterial Kit (RBC Bioscience, New Taipei City, Taiwan). The Nextera XT DNA library preparation kit (Illumina, San Diego, CA, USA) was used to prepare isolate sequencing libraries. Subsequent short-read sequencing was performed using Illumina MiSeq sequencing (Illumina, San Diego, CA, USA) according to the manufacturer’s instructions to produce 2 × 250 bp paired-end reads on DNA prepared with the MiSeq Reagent Kit v3 (Illumina). In parallel, long-read sequencing was performed using an Oxford Nanopore Technology MinION equipped with an R9.4.1 flow cell and the SQK-RBK004 rapid barcoding kit. Guppy (v6.4.6) was then used with the dna_r9.4.1_450bps_sup.cfg configuration file for basecalling in super high accuracy mode and demultiplexing.

Trimmomatic (v0.38) was used to trim the Illumina MiSeq reads with the following options: “LEADING” set to 10, “TRAILING” set to 10, “SLIDINGWINDOW” set to “4:20”, “MINLEN” set to 40, and “ILLUMINACLIP” set to “NexteraPE-PE.fa:2:30:10” [28]. The quality of the trimmed reads was checked using FastQC (v0.11.7) [29]. Hybrid assemblies were generated following the recommendations of Wick et al. [30] for automating the generation of long-read first hybrid assemblies. Briefly, long reads were trimmed using Filtlong (v0.2.0) (https://github.com/rrwick/Filtlong, accessed on 5 May 2021) and used to generate de novo assemblies using Flye with the “--genome-size” parameter set to 6,230,593, and the other parameters were left at their default values (v2.9.1) [31]. The resulting assemblies were polished using Medaka (v1.7.3) (https://github.com/nanoporetech/medaka, accessed on 1 June 2023) with the ‘r941_min_sup_g507’ model, followed by Polypolish (v0.5.0) [32] and POLCA (v4.1.0) [33]. BWA (v0.7.17) (Li and Durbin, 2009) [34] was used to map the reads to the draft assemblies using the default options, with forward and reverse reads mapped separately.

SNPs and indels were detected using Snippy (v4.6.0) (https://github.com/tseemann/snippy, accessed on 3 August 2023) and Samtools (v1.13) [34] with the Bakta (v1.8.2) [35] annotated reference genome hybrid assembly and the trimmed short-reads as inputs. Detected variants were confirmed by manual investigation in IGV [36]. Large structural deletions were detected by generating coverage plots of the short-reads using tinycov (v0.4.0) (https://github.com/cmdoret/tinycov, accessed on 17 April 2023) on the BAM files generated by Snippy. Mummerplots (v3.5) [37] was used to confirm the large chromosomal deletions and to search for other large insertions and inversions by comparing the variant hybrid assemblies to the reference hybrid assembly (i.e., the genome of the original strain before selection). Pan-genome analysis was performed on the hybrid assemblies using Prokka (v1.14.6) [38] and Roary (v3.13.0) [39] with the “—mafft” option enabled, the minimum identity set to 95%, and paralogs not split (“-s”). Data manipulation and visualization for the circular chromosomic view used R (v4.2.2) and the tidyverse (v1.3.2) [40] and circlize (v0.4.15) [41] packages.

*E. coli* sequencing was performed as follows: Total genomic DNA was extracted from the *E. coli* isolates using the DNeasy UltraClean Microbial kit (Qiagen, Hilden, Germany). The DNA was prepared for long-read sequencing (Rapid barcoding kit SQK-RBK114.24, Oxford Nanopore Technology, Oxford, UK) on a MinION equipped with an R10.4.1 flowcell. Basecalling of the nanopore data was carried out using Guppy (v6.3.8) [13] in super high-accuracy mode, followed by demultiplexing also with Guppy. The reference genome was assembled using Unicycler [42] (v0.5.0) [43] and annotated using Prokka [38] (v1.14.6). SNP calling was carried out with Snippy (https://github.com/tseemann/snippy, accessed on 3 August 2023) (v4.6.0) using the reads of the variants against the annotated reference genome. Coverage was visually inspected using UGene (v44.0) [44] after mapping the reads on the reference with Bowtie2 (v2.5.0) [45].

### 2.8. ELISA Mucin Quantification Assays

Quantitative ELISA tests were performed on GOAC triplicates to assess their mucin secretion profiles based on the content of the egressing culture medium coming out of their outlet catheter. Three triplicates of three different cell compositions were analyzed: 100% Caco-2, 50%/50% Caco-2–LS 174T, and 100% LS 174T. Standards, controls, and samples were obtained in technical duplicates. All GOACs were analyzed 120 h after cell seeding: after this delay, GOACs’ egressing fluid was collected at a pumping speed of 1.0 mL/h for 30 min (volume of 0.5 mL required for the assay) and centrifuged at 2000× *g* for 10 min to remove debris, and then stored at −20 °C. Both protocols were then carried out according to the manufacturer’s instructions considering the following points: For the Human MUC2 SimpleStep ELISA^®^ Kit (ABCAM ab282871, Cambridge, UK), the samples were diluted at a 1:2 ratio into Sample Diluent solution. For the Human Mucin 5AC SimpleStep ELISA^®^ Kit (ABCAM ab303761), the samples were diluted at a 1:20 ratio into Sample Diluent solution. The optical density measurements were recorded at 450 nm during 1 s by well. The analysis was performed by subtracting the average blank control standard absorbance value from all other absorbance values. Four-parameter logistic (4PL) curve fit was created by plotting the absorbance values for each standard concentration plotted on log–log axes. The concentration of the target protein in the sample was then determined considering the sample dilution factor. Mean comparison between tested groups was obtained by univariate ANOVA using SPSS (IBM SPSS Statistics for Windows, Version 27.0. IBM Corp., Armonk, NY, USA). Post hoc multiple paired comparisons were generated after Bonferroni correction.

## 3. Results

### 3.1. GOAC Cell Culture

**Figure 2:**
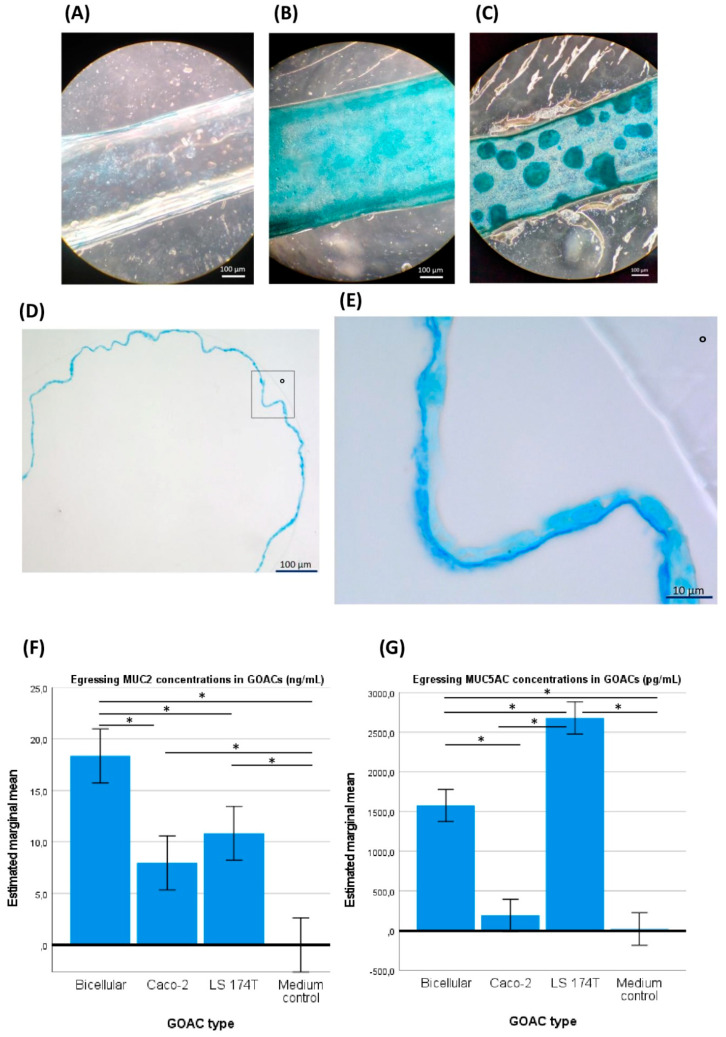
GOAC visualization and mucus production. (**A**) An upper view in light microscopy of a negative control GOAC (seeded with the usual cell load but without prior extra-cellular matrix coating) shows little to no staining after in-GOAC Alcian blue staining protocol compared to a fully confluent 100% Caco-2 cellularized GOAC (**B**) and a 50%/50% Caco-2–LS 174T bicellular GOAC (**C**). A general view (**D**) and close-up view (from the rectangle in D, ° as graphical marker) (**E**) of a histological section performed in a cellularized segment of a 100% Caco-2 GOAC’s inlet catheter add further validation of the model’s inner tissue architecture. A continuous mono-layer epithelium is seen lining the inner plastic wall (°) of the inlet catheter, seemingly producing a continuous ~2 µm thick layer of an intensely Alcian blue-stained compound, likely mucus. Quantitative ELISA tests targeting mucin MUC2 (**F**) reveals significantly superior MUC2 production in bicellular GOACs than in both the medium negative control and any mono-cellular GOACs. An identical assay targeting mucin MUC5AC (**G**) reveals significantly superior MUC5AC production in the bicellular GOACs than in the medium negative control and in the Caco-2 mono-cellular GOACs but significantly inferior MUC5AC production than in the LS 174T mono-cellular GOACs. *: statistically significant difference (*p* < 0.05) in univariate ANOVA (see Section 2).

Our first GOAC series was initially seeded with Caco-2 cells only. To visually validate their viability and their aptitude for parietal mucus secretion, direct in-GOAC Alcian blue staining was performed. The same staining was performed on a negative control chip seeded with the same Caco-2 load but without prior ECM coating of the GOAC, theoretically preventing cell adhesion, growth to confluence, and parietal mucus secretion. Light microscopy through the GOAC’s fully transparent PDMS and glass scaffold allows for a direct upper view of these stained GOAC channels (Figure 2A,B). Furthermore, by performing the in-GOAC Alcian blue staining protocol, we serendipitously discovered that some segments of the stained GOAC’s inlet catheters remained similarly stained and were thus suspected to have been cellularized during the ECM coating and cell seeding processes. While the glass–PDMS scaffold of the actual GOAC channel was not suited for microtome sectioning, these catheter segments were deemed microtome-compatible. To further validate the developed model, transverse histological sections were performed in one of these catheters, confirming the hypothesis that the Caco-2 mono-culture achieved circumferential epithelialization of the catheter’s inner walls, displaying a continuous ~2 µm thick apical strip of intensely Alcian blue-stained material, likely to be an apical mucus layer (Figure 2D,E).

When the ulterior GOACs were instead seeded with a 50%/50% mix of Caco-2 and LS 174T cells (“bicellular GOACs”), we similarly showed the ability of these cell lines to co-exist in cultured GOACs (Figure 2C). To validate the functional maturity of these GOACs, two quantitative ELISA tests specifically targeting mucins MUC2 and MUC5AC, respectively, were performed on egressing culture medium of three triplicates of GOACs (100% Caco-2; 100% LS 174T; 50%/50% Caco-2–LS 174T) and on FULL medium as a negative control. The results suggest that the bicellular GOACs produced an optimal compromise in the MUC2-MUC5AC secretion profile compared to the mono-cellular counterparts. MUC2 titers were indeed significantly higher in the bicellular GOACs than in the 100% Caco-2 (univariate ANOVA *p*-value < 0.001) or 100% LS 174T (*p* = 0.01) GOACs. MUC5AC titers were significantly higher in the bicellular GOAC models than in the 100% Caco-2 GOACs (*p* < 0.001), but lower than in the 100% LS 174T GOACs (*p* < 0.001) (Figure 2F,G).

### 3.2. GOAC Bacterial Infection Outcomes

We started our first infection series with *P. aeruginosa* CN573. At this stage, we were still culturing GOAC series seeded either with 100% Caco-2 cells or with 50%/50% Caco-2–LS 174T cells. Therefore, several infection conditions tested with *P. aeruginosa* were compared in both GOAC types. This was not the case during our later infection series with *E. coli* ST131, which focused solely on bicellular GOACs.

**Figure 3:**
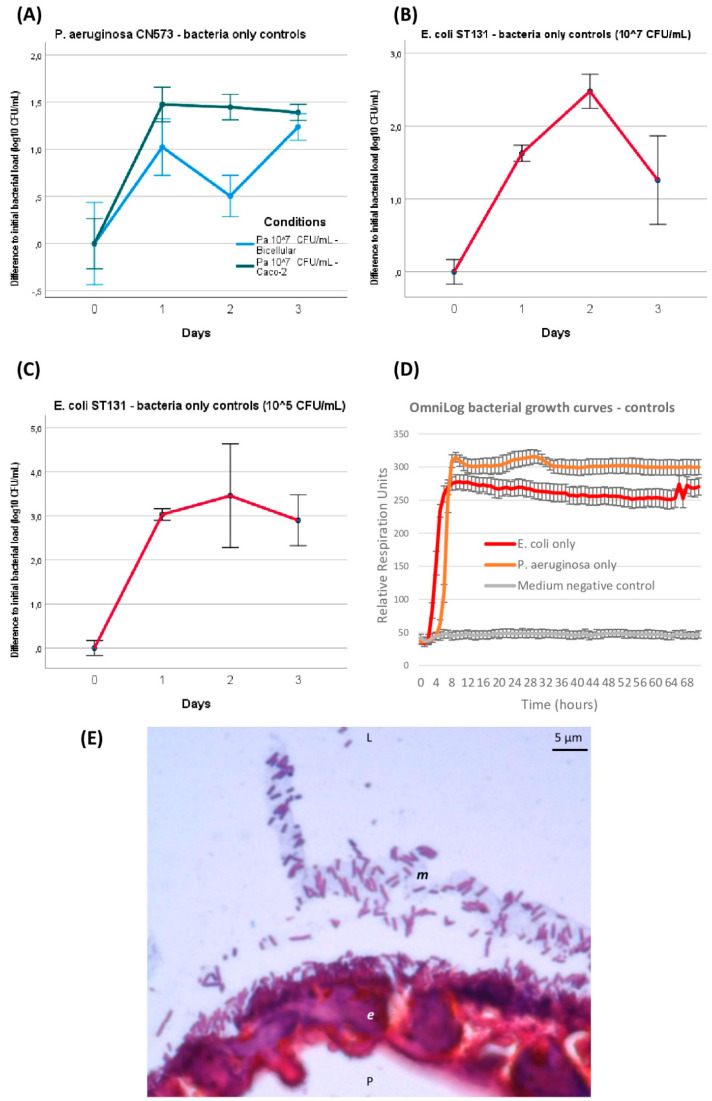
**GOAC and OmniLog bacteria-only control conditions.** Bacterial titers’ evolution in GOACs when infected with *P. aeruginosa* CN573 (10^7^ CFU/m; n = 3) (**A**) or *E. coli* ST131 at either higher (10^7^ CFU/mL; n = 3) (**B**) or lower (10^5^ CFU/mL; n = 3) initial titer concentrations (**C**). Bacterial control growth curves in static in vitro conditions using OmniLog automated incubator, with bacterial respiration units being measured as proxy for bacterial growth (n = 16 wells for each condition; initial bacterial load of 10^5^ CFU/well; error bars represent +/−1 standard deviation of mean) (**D**). Microscopic view of GOAC inlet catheter transversal histological section after 48 h *P. aeruginosa* CN573 10^7^ CFU/mL colonization after Gram staining. *P. aeruginosa* appear as numerous typical Gram-negative pink-red bacili that seemingly form homogeneously adherent lining to apical pole of continuous Caco-2 GOAC epithelium (e), and can also be seen forming consortium in likely apical mucus lining (m, grey). (**E**) Microscopic view of GOAC inlet catheter transversal histological section after 48 h *P. aeruginosa* CN573 10^7^ CFU/mL colonization after Gram staining. *P. aeruginosa* appear as numerous typical Gram-negative pink-red bacilli that seemingly form homogeneously adherent lining to apical pole at continuous Caco-2 GOAC epithelium (*e*) and can also be seen forming consortium in likely apical mucus lining (*m*, grey). *[Pa: P. aeruginosa; CFU: colony forming unit; L: luminal; P: parietal]*.

GOAC infection and outcome follow-ups over 72 h were performed. When introduced alone at 10^7^ CFU/mL, without adding any phage over 72 h, *P. aeruginosa* and *E. coli* infection in the GOACs followed similar dynamics: on a log10 scale, the bacterial titer egressing from the GOAC significantly increased by approximately onefold at 24 h regardless of the tested condition (Figure 3A–C). The bacterial titer then remained stable on a plateau around 10^8^ CFU/mL at 48 h and 72 h for *P. aeruginosa* in the 100% Caco-2 GOACs. Comparatively, the *P. aeruginosa* titer mildly decreased at 48 h in the bicellular GOACs but reached similar titers as both conditions again at 72 h (Figure 3A). On the contrary, *E. coli* showed a temporary spike in bacterial growth at 48 h. A similar assay with a lower initial bacterial load of 10^5^ CFU/mL, used as control conditions for preventive assays, resulted in the same dynamics with a similar 10^8^ CFU/mL titer at 24 h, remaining stable afterwards (Figure 3C). These series of bacterial infections served as control conditions for phage assays in the next GOAC series.

Furthermore, these in-GOAC bacterial infection dynamics seemed similar to those observed over 72 h in an OmniLog automated incubator, serving as a control in in vitro growth conditions. Indeed, a logarithmic phase of bacterial growth was systematically completed after less than 24 h of incubation, and a stable plateau was maintained until final read at 72 h (Figure 3D). Co-existence and expected adherence of inoculated bacteria to the apical side of the epithelium, and likely inside its secreted mucus, were observed on Gram-stained histological sections after 48 h of 10^7^ CFU/mL *P. aeruginosa* inoculation (Figure 3E).

### 3.3. P. aeruginosa CN573 versus Phage PNM

**Figure 4:**
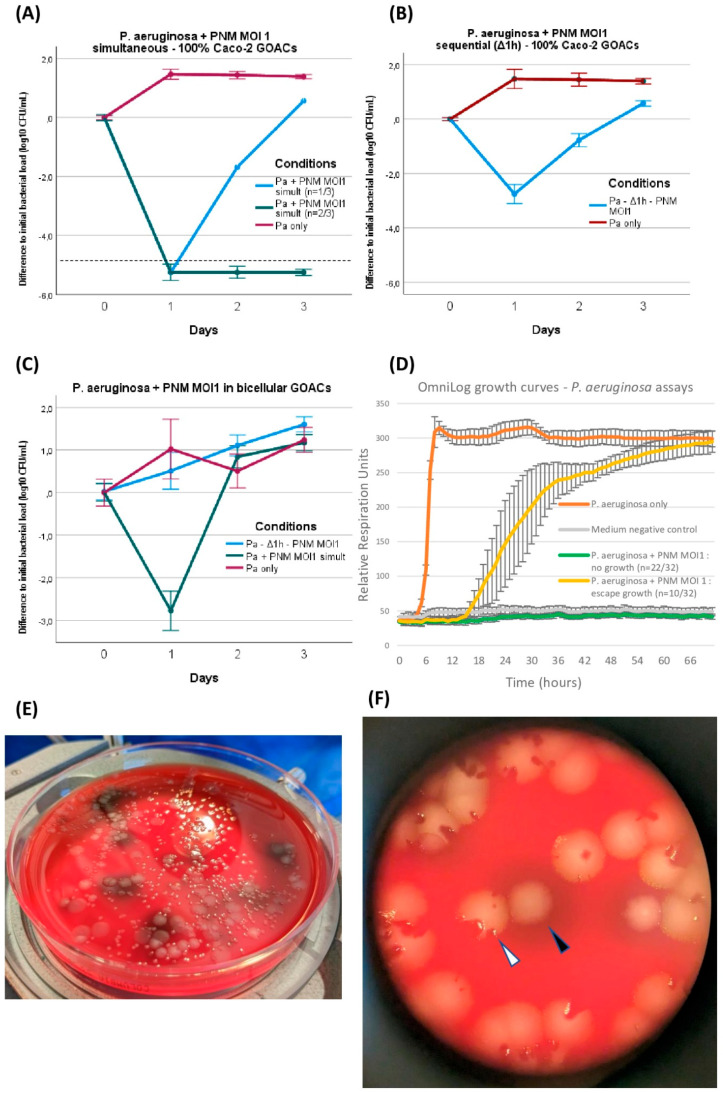
***P. aeruginosa* CN573 versus phage PNM assays.** *P. aeruginosa* titer evolution when introduced in GOACs with phage PNM (MOI = 1) in various conditions: (**A**) simultaneous introduction of *P. aeruginosa* and PNM in 100% Caco-2 GOACs (dotted line represents detection threshold in spread plating, <10^2^ CFU/mL) (n = 3), (**B**) sequential introduction of *P. aeruginosa* first with subsequent introduction of phage PNM after 1 h static incubation delay in 100% Caco-2 GOACs (n = 3), (**C**) replication of these two conditions in bicellular GOACs (n = 3 each). (**D**) *P. aeruginosa* and PNM (MOI = 1) control growth curves in static in vitro conditions using OmniLog automated incubator, with bacterial respiration units being measured as proxy for bacterial growth (initial bacterial load of 10^5^ CFU/well; n = 32 wells for *P. aeruginosa* + PNM, 16 wells for *P. aeruginosa* only, and 16 wells for negative control; error bars represent +/−1 standard deviation of mean). (**E**) Spread plating on 5% sheep blood Columbia agar plates highlights intense phenotypic diversification in *P. aeruginosa* CN573 after co-evolution with phage PNM in GOACs, displaying at least four distinct colony morphologies out of single GOAC after 48 h. (**F**) Close-up view of one of these plates reveals co-existence of wild-type colonies (white arrow), systematically including phage-induced lysis plaques, along with modified colonies, for example, exhibiting dark pigmented halo (black arrow), systematically devoid of any lysis plaques. *[Pa: P. aeruginosa; GOAC: Gut-On-A-Chip; MOI: Multiplicity of Infection]*.

The joint introduction of both *P. aeruginosa* and its corresponding phage PNM was analyzed under different conditions in both Caco-2 GOACs and bicellular GOACs. The only tested MOI was 1. On at least one out of three timepoints (24, 48, and 72 h), all tested conditions differed significantly from the aforementioned control GOACs infected with the same *P. aeruginosa* load without phage, though to different extents (Figure 4).

*P. aeruginosa* and PNM were first mixed at MOI 1 immediately before manual instillation in 100% Caco-2 GOACs and restart of the pumping system. This led to a marked reduction in bacterial titers inside the GOACs, with all three GOACs of the triplicate reaching below the bacterial detection threshold at 24 h (<10^2^ CFU/mL). One of these two GOACs then reconstituted a detectable bacterial population, eventually surpassing the initial instilled bacterial titer at 72 h only. The other two GOACs remained at undetectable bacterial titers for 72 h, suggesting sterility (Figure 4A).

We then introduced these same bacterial and phage loads inside a similar triplicate of 100% Caco-2 GOACs, but this time by first instilling *P. aeruginosa* alone and letting it statically incubate in the GOACs in a 37 °C/5% CO_2_ incubator for one hour before introducing the same phage load (MOI = 1). Again, a reduction in bacterial titers was observed in all three GOACs, but to a lesser extent compared to the previous condition of simultaneous administration. Indeed, none of the GOACs reached bacterial undetectability at any timepoint, and all three had grown back to surpass the initial bacterial titer concentration at 72 h (Figure 4B).

Replicating both of these conditions in bicellular GOACs instead of Caco-2 GOACs led to even more bacterial-favoring outcomes, with all other factors being equal. Simultaneous phage–bacteria introduction again led to a significant reduction in bacterial titers at 24 h, but no GOAC ever reached bacterial undetectability, contrasting with their 100% Caco-2 counterparts. In addition, the bacterial titers surpassed the initial instilled titers at 48 h. Sequential administration after a similar one-hour delay after bacterial incubation was this time unable to induce any reduction in the initial instilled bacterial titers at 24 h. At 72 h, both of these conditions’ bacterial titers had reached similar levels as their bacteria-only control GOACs (Figure 4C).

During these assays, bacterial growth or regrowth despite phage lytic pressure was accompanied by the emergence of phenotypic variants of *P. aeruginosa*, identified on a visual basis of modified colony morphology on blood agar plates (Figure 4E,F). All of these variants were confirmed to belong to the *P. aeruginosa* species by MALDI-TOF. These variants’ morphotypes were reproductible between GOAC replicates in which both *P. aeruginosa* and PNM were introduced, but not in any of the bacteria-only control GOACs. This led us to suspect a non-random selection of a significant fitness advantage correlated to these phenotypic alterations, including, for example, the de novo acquisition of bacterial phage resistance (BPR) mechanisms, allowing for bacterial regrowth. To test this hypothesis, these variants were subcultured on blood agar plates, and the phage PNM’s EOP was assessed anew on each of them compared to the initial *P. aeruginosa* CN573 strain. The results confirm the emergence of BPR in all variants (Table 1).

**Figure 5:**
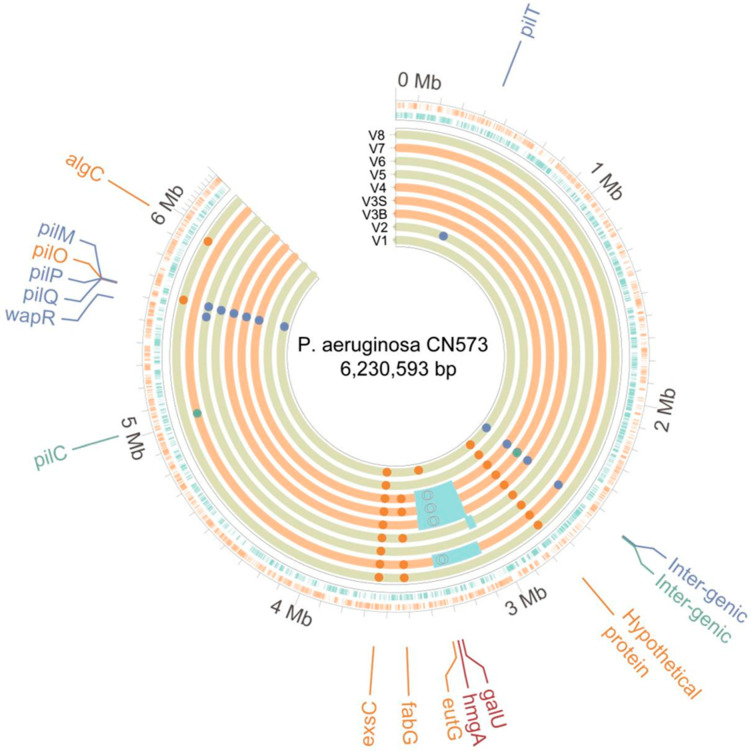
A circular chromosomic view (CCV) of the bacterial genomes of the nine sequenced *P. aeruginosa* variants. The first two rings (starting from the outside to the inside) indicate the coding regions on the plus and minus strands, and the nine inner rings show the genomic variations in the partially phage PNM-resistant V1 and V2 isolates and the fully resistant V3B, V3S, V4, V5, V6, V7, and V8 isolates. The orange inner rings indicate the four brown-colored variants. Small deletions are highlighted in dark blue, insertions are highlighted in green, and SNPs are highlighted in orange. The light blue boxes highlight the large chromosomal deletions detected in some of the variants. The position of the *galU* and *hmgA* genes, associated with the brown phenotype, are shown in burgundy. [*bp: basepairs; Mb: megabases; SNP: single-nucleotide polymorphism]*.

These bacterial variants were then sequenced to investigate the potential genomic basis for these phenotypic modifications. Consistent with the identification of total or partial BPR phenotype for each of them, genomic variations likely to cause these modified phenotypes were identified for each of them. All nine sequenced variants showed a variety of single-nucleotide polymorphisms (SNPs) and indels in genes coding for *P. aeruginosa*’s type IV pilus (T4P) complex (*pilC*, *pilM*, *pilO*, *pilP*, *pilQ*, and *pilT*), which is known to be a key surface receptor for phage PNM’s infectivity and has already been identified as a BPR driver in clinical cases of anti-*P. aeruginosa* phage therapy [13,46] (Table 1, Figure 5). Some of these variants also showed other SNPs and indels in genes of other complexes, such as the type III secretion system (*exsC*) as well as membrane lipids and LPS biosynthesis (*algC*, *fabG*, and *wapR)*. A synonymous variant in a putative protein located next to (and suspected to be part of) the type VI secretion systems (T6SS) (MLIPEN_12965) was also detected. Lastly, five variants contained large chromosomal deletions ranging from 46 kb to 330 kb, including four brown-colored variants which were systematically and specifically found to harbor structural chromosomal deletions including the *galU* and *hmgA* genes (Table 1, Figure 5).

We also tried to compare the rate of emergence of these bacterial growths under phage pressure to a more neutral in vitro assay. *P. aeruginosa* CN573 and phage PNM were thus incubated in 96-well microplates in an automated OmniLog incubator, with bacterial growth being measured every 20 min by tetrazolium dye reduction assay. For the sake of comparison to GOAC conditions, the MOI, temperature, and duration of incubation were the same (1, 37 °C, and 72 h, respectively). Bacterial “escape” growth despite phage pressure was less frequent in these OmniLog assays than in the GOACs, with most OmniLog replicates (22/32) not showing detectable growth over 72 h (Figure 4D). This differs significantly from the bicellular GOACs’ dynamics in both simultaneous and sequential infection conditions (Fischer’s exact test *p*-value = 0.0437) and from the Caco-2 GOACs in sequential infection condition (*p* = 0.0437), but it does not differ from the Caco-2 GOACs under a simultaneous infection condition (*p* = 1).

### 3.4. E. coli ST131 versus Phages ES17 and HP3

Similar assays were conducted to investigate infection dynamics between *E. coli* ST131 and the two anti-*E. coli* lytic phages ES17 and HP3. For these *E. coli* assays, only bicellular GOACs were used.

**Figure 6:**
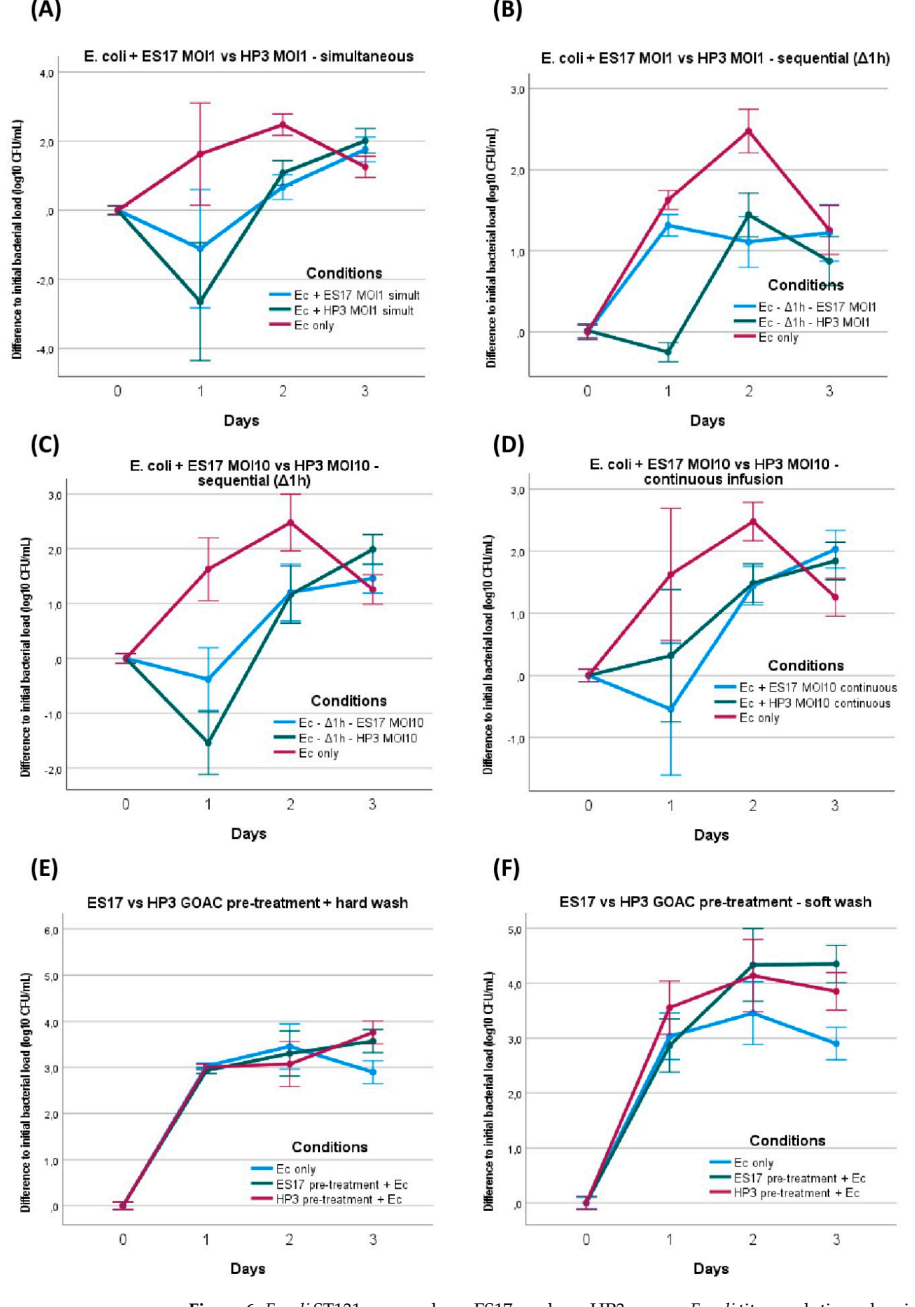
*E. coli* ST131 versus phage ES17 or phage HP3 assays. *E. coli* titer evolution when introduced in bicellular GOACs with either phage ES17 or phage HP3 in various conditions: (**A**) simultaneous introduction of *E. coli* and either phage at MOI = 1 (n = 3 each), (**B**) sequential introduction of *E. coli* first with subsequent introduction of either phage after 1 h static incubation delay at MOI = 1 (n = 3 each), (**C**) sequential introduction of *E. coli* first with subsequent introduction of either phage after 1 h static incubation delay at MOI = 10 (n = 3 each), (**D**) introduction of *E. coli* and then continuous 72 h infusion of either phage at MOI = 10 (n = 3 each). Preventive assays were also performed to establish *E. coli* titer evolution when introduced (at lower initial titer concentration of 10^5^ CFU/mL instead of usual concentration of 10^7^ CFU/mL) in bicellular GOACs pre-treated with either phage at titer concentration of 10^8^ PFU/mL by letting phage pre-treatment statically incubate in GOACs for 1 h and then submitting GOACs to either hard (**E**) or soft (**F**) washing protocol before *E. coli* introduction (n = 3 each). *[Ec: E. coli; GOAC: Gut-On-A-Chip; MOI: Multiplicity of Infection]*.

*E. coli* was first mixed at MOI 1 with phage ES17 or phage HP3 immediately before manual instillation in bicellular GOACs and the restart of the pumping system. Compared to the bacteria-only control GOACs, this only induced a statistically significant reduction in the bacterial titer at 24 h with HP3, but not with ES17. At 48 h, both the ES17- and HP3-treated GOACs had a lower concentration of bacterial titers than the controls, though both were also significantly higher than the initially instilled bacterial titer levels. At 72 h, both conditions had bacterial titer levels similar or superior to the control GOACs (Figure 6A).

We then introduced these same bacterial and phage loads inside a similar triplicate of bicellular GOACs, but this time by first instilling *E. coli* alone and letting it statically incubate in the GOACs in a 37° C/5% CO_2_ incubator for one hour before introducing the same phage load (MOI 1) of either ES17 or HP3. Similar to the *P. aeruginosa* assays, this led to more bacteria-favoring outcomes, with all other factors being equal. The HP3-induced relative reduction in bacterial titers at 24 h, while still statistically significant, was less marked. ES17 failed to even maintain the status quo compared to the initially instilled bacterial titer levels at 24 h. By 72 h, both conditions had reached similar bacterial titer levels to the bacteria-only controls (Figure 6B).

We decided to replicate this condition of sequential administration using a tenfold higher phage load (MOI 10 instead of 1 for all previous assays), keeping all other parameters unchanged. This led to a moderately more favorable phage effect, seemingly reverting the bacteria-favoring effect of sequential administration instead of simultaneous administration. The titer curves are indeed largely similar in these conditions to those of the GOACs tested with simultaneous bacteria–phage introduction. Yet even this higher MOI failed to elicit a major reduction in bacterial titers below the detectability threshold at any timepoint. In addition, ES17 again seemed to perform more poorly than HP3 in these conditions, failing to reduce the bacterial titer levels below the initially instilled bacterial titer levels at any timepoint (Figure 6B).

We then investigated whether phage efficacy in these GOACs could be improved by administering them in a continuous way instead of through a single initial introduction of a given phage load. To test this, syringes loaded with NAB medium in the alimentation pump were supplemented with MOI 10 titers of either ES17 or HP3, ensuring the continuous top-down infusion of both phages through the whole length of the model for the whole 72 h of the assay. This did not lead to significantly better phage outcomes, with bacterial titers never reaching sub-detectability levels at any timepoint, surpassing the initially instilled bacterial titer levels at 48 h and even slightly surpassing the bacteria-only control titers at 72 h. However, for the first time, ES17 seemingly performed slightly better than HP3 at 24 h, achieving significantly lower bacterial titer levels than the bacteria-only control as opposed to HP3 (Figure 6D).

In the next assays, we wanted to investigate the potential preventive properties of these two phages rather than their curative ones. As opposed to the previously investigated “sequential” condition where bacteria introduction precedes phage introduction, here, we pre-treated bacteria-free GOACs with phages, and then introduced bacteria and analyzed whether phage pre-treatment could prevent bacterial settlement in the GOACs. The GOACs were pre-treated with phages and then washed with two different washing protocols (see the Section 2). Bacterial introduction to the model was then attempted by infusing *E. coli* in a continuous flow, similar to the previous assay; syringes loaded with NAB medium in the alimentation pump were supplemented with a lower titer concentration of 10^5^ CFU/mL, and they were then infused through the GOACs at a usual rate of 120 µL/h. The control GOACs in these conditions were thus also seeded with 10^5^ CFU/mL of *E. coli* instead of the usual 10^7^ CFU/mL concentration in all other GOAC assays. Despite evidence of phage persistence in the pre-treated GOACs through the observance of lysis plaques on the output CFU in all four conditions, neither of these pre-treatment protocols managed to prevent bacterial settlement and growth in these GOACs, with none of the bacterial titers measured being inferior to their bacteria-only controls at any timepoint (Figure 6E,F).

**Figure 7:**
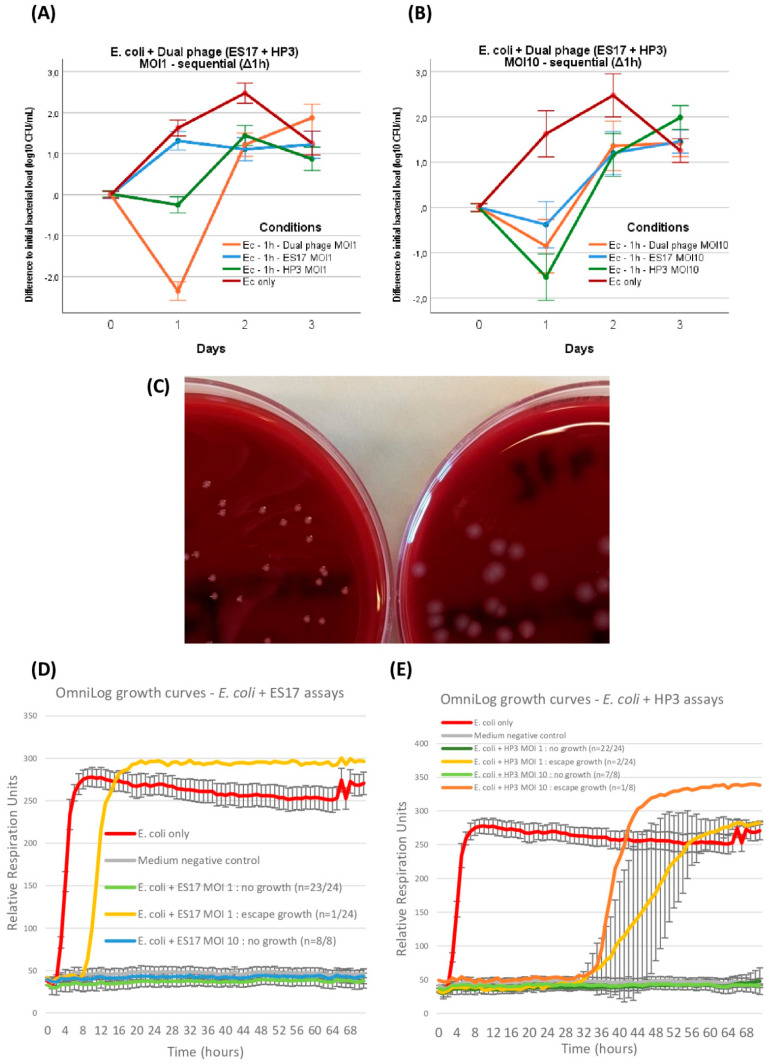
*E. coli* ST131: dual phage and OmniLog assays. *E. coli* titer evolution when introduced in bicellular GOACs with 50%/50% combination of phage ES17 and phage HP3 in comparison with both mono-phage counterparts at MOI = 1 (n = 3 each) (**A**) or MOI = 10 (n = 3 each) (**B**). Example of modified *E. coli* ST131 colony morphology (left, variant ST131-V1 see Table 2) after exposition to HP3 at MOI = 1 compared to wild-type colony morphology (right) (**C**). *E. coli* with either ES17 (**D**) or HP3 (**E**) control growth curves in static in vitro conditions using OmniLog automated incubator, with bacterial respiration units being measured as proxy for bacterial growth (initial bacterial load of 10^5^ CFU/well; n = 24 wells for each MOI = 1 assay, 8 wells for each MOI = 10 assay, 16 wells for *E. coli* only, and 16 wells for negative control; error bars represent +/−1 standard deviation of mean). *[Ec: E. coli; MOI: Multiplicity of Infection]*.

Up to this point, the ES17 and HP3 phages had only been tested separately with the aim of comparing their respective efficacy, or lack thereof, at modifying in-GOAC bacterial dynamics. Next, we wanted to investigate whether these phages seemed to have synergistic potential in this model. Accordingly, we replicated the sequential administration series by first incubating *E. coli* alone in GOACs and then introducing a 50%/50% mix of ES17 and HP3 phages (combined MOI of 1 and 10 respectively). At MOI 1, this assay generated significantly better phage outcomes at 24 h than either mono-phage MOI 1 counterparts in the same conditions. This was not the case when combining MOI 10, which unexpectedly performed worse than MOI 1 at 24 h, though still managing to temporarily reduce bacterial titer levels below initially instilled bacterial titer levels, but not to a greater extent than HP3 or ES17 alone at MOI 10 (Figure 7A,B).

Following a similar approach to the *P. aeruginosa* assays, the development of thriving bacterial growth under various conditions of phage pressure in these GOACs led us to investigate potential underlying causes. In multiple independent GOACs, the occasional observation of phenotypic modifications in *E. coli* colony morphologies led us to postulate a fitness advantage in these variants, which is possibly correlated to BPR acquisition (Figure 7C). Similarly, these variants were subcultured on blood agar plates, and then the respective EOPs of phages ES17 or HP3 were assessed again on each of them, according to the phage they had previously been exposed to. As opposed to *P. aeruginosa*, though, only one variant (1/5) displayed a mildly reduced EOP, with the majority (4/5) not displaying any modifications in phage susceptibility. The long-read sequencing of these variants did not highlight any genomic modification in line with the described BPR-acquisition pathways, only highlighting one deletion causing a non-synonymous frameshift variation in a hypothetical protein in one ES17-induced variant (Table 2).

Similar to *P. aeruginosa* assays, OmniLog growth assays in similar conditions yielded significantly lower rates of bacterial “escape” growth over the same timeframe compared to GOACs, this time for both phages either at MOI 1 (ES17: *p* = 0.0014; HP3: *p* = 0.0034) or at MOI 10 (ES17: *p* = 0.0061; HP3: *p* = 0.0242) (Figure 7D,E).

### 3.5. Serum-Supplemented OmniLog Assays

To maximize comparability, the above-mentioned “control” OmniLog assays were eventually replicated with the modification of a single parameter: the LB culture medium was supplemented with 20% FBS to reach identical FBS proportions as the medium infused in GOACs.

**Figure 8:**
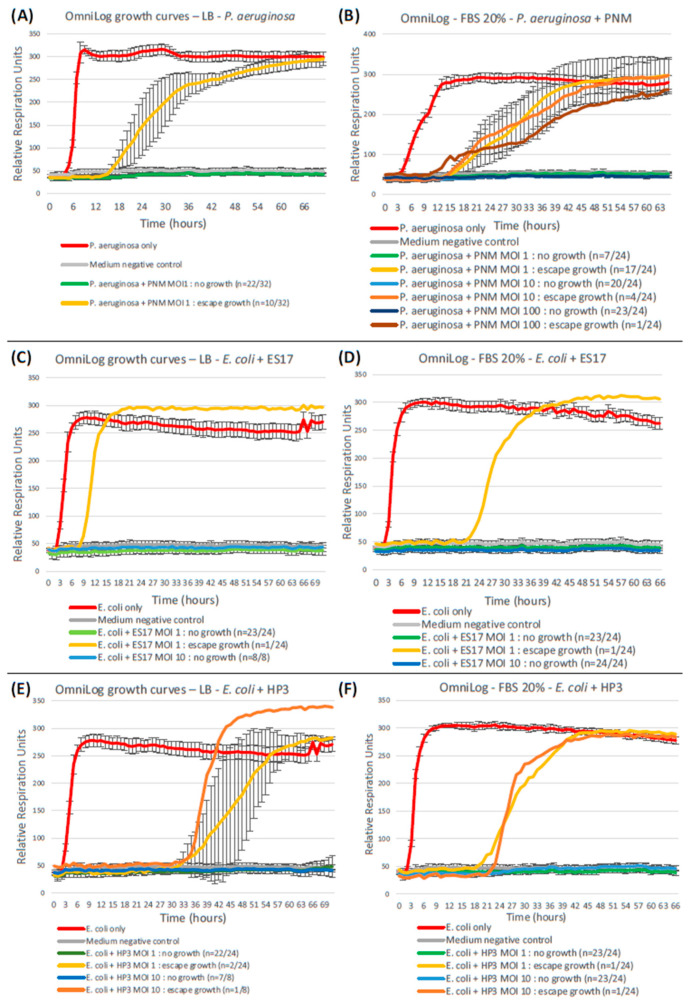
Serum-supplemented OmniLog assays. Replicating previous OmniLog assays with the supplementation of 20% fetal bovine serum (the same proportion as in the GOAC culture medium) to LB yields significantly higher bacterial escape growth rates in *P. aeruginosa*–PNM assays (**A**,**B**), but not in *E. coli*–ES17 (**C**,**D**) nor in *E. coli*–HP3 (**E**,**F**) assays.

A comparison between these three pairs of assays (i.e., *P. aeruginosa* CN573 and PNM, *E. coli* ST131 and ES17, and *E. coli* ST131 and HP3) is presented in Figure 8.

Qualitatively, FBS supplementation does not seem to significantly modify the escape growth profile, still starting at least six hours after the bacteria-only positive control but eventually reaching a similar plateau by the end of the 72 h period.

Quantitatively, on the other hand, FBS supplementation leads to a significantly higher rate of bacterial escape growth than in its FBS-free counterpart when considering the *P. aeruginosa*-PNM duet (MOI 1: *p* = 0.0064), but not in the *E. coli*-ES17 (MOI 1: *p* = 1; MOI 10: *p* = 1) nor the *E. coli*-HP3 (MOI 1: *p* = 1; MOI 10: *p* = 0.443) assays. As opposed to standard FBS-free OmniLog assays, the FBS-supplemented OmniLog outcomes for *P. aeruginosa*-PNM (MOI 1) in terms of the escape growth rate thus do not differ significantly from those of the GOACs (*p* = 0.545). FBS supplementation’s deteriorating effect on PNM outcomes at MOI 1 in these OmniLog assays can, however, be surpassed by the use of higher MOIs like 10 and 100 (Figure 8).

## 4. Discussion

This work investigated bacterial dynamics under phage pressure in GOACs under numerous culture conditions focusing on two bacterial species: *P. aeruginosa* and *E. coli*. The choice to focus on these two species is based on experimental and clinical relevance, taking inspiration from our clinical practice in *Cliniques universitaires Saint-Luc* ([CUSL]-Brussels, Belgium). In CUSL, one of the most important subsets of patients concerned with MDR bacteria asymptomatic carriage and potential associated infections are pediatric liver transplant recipients; their high frequency of MDR bacteria carriage can be explained in part by the diverse geographic origins of these patients [47]. Besides its high carrier rate, this population is also especially vulnerable to “carriage–infection conversion” as the liver transplantation they endure automatically induces the three aforementioned risk factors that facilitate it: major abdominal surgery (liver transplantation surgery itself), intensive care unit stay, and immune suppression (related to anti-rejection drugs) [8]. On the one hand, *P. aeruginosa*, while not the most frequently encountered carriage in the CUSL subpopulation, has led, on occasion, to devastating carriage–infection conversions, which are responsible for high morbidity and limited therapeutic options [13]. Furthermore, its carriage rate appears as one of the most frequent ones in another large comparable series of pediatric liver transplant recipients in Korea, advocating for a variable clinical relevance according to local epidemiology [48]. Lastly, previous works have illustrated that *P. aeruginosa* fits the carriage–infection conversion paradigm [49,50]. On the other hand, choosing to work with *E. coli* appeared locally relevant given its high carriage incidence in the CUSL subpopulation. Moreover, this choice gave us access to a pair of corresponding anti-*E. coli* phages whose respective properties were recently extensively described, particularly their MBPs or lack thereof [16]. This made the *E. coli* bacteria–phage duet an ideal candidate to transition this clinically inspired problematic research model to a preliminary digestive research model such as the GOAC model.

Green and colleagues’ findings from the comparative oral administration of two phages harboring MBPs (phage ES17) or without MBPs (phage HP3) led them to conclude that the former was more efficient at lysing *E. coli* ST131 in the digestive tracts of mice. They also showed that this difference was likely due to the inhibitory (or at least bacteria-protective) effect exerted by mucins on phages devoid of MBPs as opposed to their enhancing effect on a phage harboring MBPs. In this case, the MBPs of phage ES17 seem mediated by a tail fiber protein specifically binding to heparan sulfate proteoglycans, which are ubiquitous glycoproteins present at the basement and surface membranes of cells. Their findings are consistent with a previous paradigm proposed by Barr and colleagues, the “Bacteriophage Adherence to Mucus” (BAM) model [51]. The BAM paradigm implies that some phages constitutive of animal commensal microbiomes can reach a state of prosperity in parietal mucus-rich interfaces by harboring MBPs. In the case of BAM, these MBPs have been described as being mediated by several subtypes of immunoglobulin-like domains [51,52,53,54,55]. This prosperity allows for more likely phage–bacteria encounters in these mucus-rich interfaces, inducing bactericidal properties constitutive of a “non-host-derived immunity”, for example, which is able to prevent bacterial translocation from the gut lumen to the bloodstream [51,53]. Except for the fact that MBPs of ES17 are not mediated by immunoglobulin-like domains, both of these groups’ conclusions coincide.

To investigate whether these conclusions could be extrapolated to GOACs, the GOACs required proper parietal mucus secretion, since it is an essential condition to the BAM paradigm. Histological sections of Alcian blue-stained inlet catheters of GOACs have suggested that sustained cell culture in our GOACs could induce the production of a thin continuous apical mucus layer. Furthermore, mucin quantification by specific quantitative ELISA tests illustrated that our GOACs produced detectable quantities of targeted mucins MUC5AC and MUC2 and that the production of MUC2 was increased in bicellular GOACs compared to both mono-cellular counterparts. We initially chose to supplement 100% Caco-2 GOACs with LS 174T cells because of LS 174T’s well-documented MUC2 secretion potential, whereas transcriptomic studies have suggested that Caco-2 cells had no significant MUC2 production in two-dimensional static culture [56]. Surprisingly, the 100% Caco-2 GOACs did not produce significantly less MUC2 than the 100% LS 174T GOACs. Besides having different outcome measurements (mRNA vs. antigen), this difference might be induced by the different culture conditions (two-dimensional static vs. three-dimensional dynamic), in line with previous reports of MUC2 secretion by Caco-2 cells in GOACs [57]. Optimizing MUC2 secretion, which was achieved in bicellular GOACs, seemed a key priority; it is indeed likely the most important mucus compound in organizing the architecture of the mucus-embedded microbiome along the gut walls [58]. For this reason, bicellular GOACs were considered the most physiologically relevant after the initial comparative series and were the only type of GOACs used in the *E. coli* assays. MUC2 shapes the parietal mucus layer into two contrasted sublayers, including an inner dense sublayer hostile to microbiological life, lining the gut epithelium’s apical surface and protecting it from direct bacterial contact. On the other hand, the outer sublayer is partly disaggregated, and its looser structure fosters thriving microbiological life. In *P. aeruginosa* assays, the relative lack of MUC2 abundance in 100% Caco-2 GOACs might explain why they did not seem as protective to bacterial prosperity as the 50%/50% Caco-2–LS 174T bicellular GOACs under the same phage conditions.

In *P. aeruginosa* assays, bacterial “escape” growth following an initial decline under phage pressure was correlated with the emergence of various *P. aeruginosa* colonies of modified morphology. We postulated that these morphological modifications were likely correlated with fitness advantages, allowing for bacterial growth under phage pressure, such as the acquisition of BPR mechanisms. This seemed especially likely given the absence of morphologically modified colonies in the bacteria-only control GOACs. Consistent with this hypothesis, PNM susceptibility testing performed with a DAO assay on these variants always illustrated a dramatic loss in phage susceptibility, which is consistent with the clinical thresholds used to qualify BPR (EOP < 0.01). Accordingly, the sequencing of these variants revealed the systematic acquisition of T4P-related genomic modification, known as causative of BPR phenotypes [13,46]. Other genomic modifications were also detected, some of which are also known to cause BPR, affecting structures and functions such as LPS biosynthesis, pyomelanin metabolism conferring the brown coloration of some variants (*hmgA*), or O-antigen-related virulence (*galU*) [59]. These alterations highlight a key limitation of GOACs since all of their associated fitness costs could be comparatively significantly higher in human hosts. First, a possible loss of virulence due to T4P-, LPS-, or *galU*-related alterations could hinder in vivo bacterial survival according to the phage-induced virulence trade-off (PIVT) paradigm [13,59]. Second, potential alterations in the T6SS could hinder in vivo fitness, especially the digestive fitness of *P. aeruginosa* given the key role played by T6SS in inter-species bacterial competition in a given ecological niche, a feature that is likely not valued in a GOAC devoid of any bacterial competition [60]. It should be noted that the synonymous variant found in this work and suspected to be part of the T6SS complex does not necessarily result in an absence of phenotypic modification regarding the T6SS structure or function since codon usage bias has been shown to potentially generate phenotypic changes between synonymous codons, including in *P. aeruginosa* [61,62]. Lastly, pyomelanogenic phage-resistant *P. aeruginosa* variants related to *hmgA* alterations are correlated to a higher susceptibility to colistin, a last-resort antibiotic against *P. aeruginosa*, suggesting possibly synergistic properties of phage–antibiotic interactions on these variants [59,63]. These mechanisms are part of an increasingly well-described potential for phage-induced trade-offs in *P. aeruginosa*, the use of which could yield interesting adjuvant therapeutic potential regardless of the phages’ lytic efficacy [64].

In *E. coli* assays, a similar approach to phenotypic variant analysis during bacterial “escape” growth did not provide similar results. As opposed to the *P. aeruginosa* assays, ES17 and HP3 susceptibility testing performed by DAO assays on these variants never showed dramatic decreases in the EOP, suggesting an absence of BPR acquisition in these variants. These results could suggest gut-specific mechanisms of *E. coli* phage resistance or phage evasion, which cannot be reproduced in phage–bacteria assays outside of the GOAC and thus can possibly not be correlated with expected BPR-inducing genomic alterations in these variants. This hypothesis goes in line with the findings of Chibani-Chennoufi and colleagues [65]. Their findings illustrate that *E. coli* existing as long-term colonizers in the guts of mice are significantly more resistant to digestive phage therapy than *E. coli* that are newly introduced into the gut, while both types of *E. coli* display similar phage susceptibility outside of the gut environment. Whether these gut-specific determinants of phage resistance were even mediated by bacteria themselves was not certain, suggesting possible mechanisms of “non-bacterial phage resistance”. Consistent with this paradigm, sequencing did not highlight any genomic modification in line with the described BPR acquisition pathways, only highlighting one deletion causing a non-synonymous frameshift variation in a hypothetical protein in one ES17-induced variant. This suggests that in-GOAC *E. coli* adaptation towards phage evasion might be based on dynamic gene regulation or even on non-bacterial determinants rather than on durable genomic alterations. This hypothesis goes in line with the recent in-depth investigation conducted by Lourenço and colleagues, illustrating the tripartite (bacteria, phage, and gut environment) nature of this significant in vitro–in vivo mismatch, highlighting the importance of dynamic gene regulation in gut-colonizing *E. coli* and its lower associated fitness costs compared to stable genomic alterations like loss-of-function mutations [66]. The same group previously illustrated that the gut’s architectural features themselves play a key role in creating “bacterial refuges” in the mucosal environment, creating conditions for long-term bacteria–phage prosperity at the spatial level regardless of the respective development of BPR and associated phage co-evolution [67]. The potential of phages to evolve along gut-specific mutational pathways has also been recently documented, including in GOAC models [24].

*P. aeruginosa* and *E. coli* assays also differ by their comparative outcomes in OmniLog assays, especially when considering serum-supplemented assays. Besides carrying serum-free OmniLog assays as “neutral” in vitro comparators, we decided to replicate them using a similar FBS proportion as that used in the GOAC infusion medium so as to rule out the contribution of serum itself in the GOAC-OmniLog outcomes’ differences. The rationale behind this is the previous description of the phage-deteriorating properties of serum, mostly reported in *Staphylococcus aureus*, though varying vastly among *S. aureus* strains [68]. These properties appear to be likely correlated with IgGs, which are thought to compete with phages for specific surface receptors, hampering phage adsorption [68,69]. Works reporting similar mechanisms in *P. aeruginosa* and *E. coli* phages are rarer, tend to illustrate these properties in phage-induced immunized serum and not in supposedly “naïve” serum, such as FBS in our case, and report potentially high variance between phages and between replicates [70,71,72]. Here, we illustrated significantly different serum-induced effects on OmniLog outcomes according to species: the effects were apparently neutral for *E. coli* and for both ES17 and HP3 phages, but significantly phage-deteriorating for *P. aeruginosa* and PNM, although this appears fully reversible with the use of higher MOIs. This represents an additional layer of analytical complexity in what fosters differences between OmniLog and GOAC outcomes, but also between GOAC and mice outcomes, as will be discussed below.

In both *P. aeruginosa* and *E. coli* assays, the sequential introduction of bacteria followed by phages after a 1 h static incubation led to more bacteria-favoring outcomes than the simultaneous introduction of the same quantities of bacteria and phages. In sequential introduction conditions as opposed to some simultaneous conditions, no GOAC ever reached undetectable bacterial titers at any timepoint regardless of the phages used and the corresponding MOI. This bacteria-favoring effect of bacterial chronological pre-existence in a digestive phage therapy model is again consistent with the aforementioned findings of Chibani-Chennoufi and colleagues [65]. This might look discouraging given that bacterial pre-existence is, by definition, present in the clinical problem that is initially tackled in this work: phage-mediated digestive decolonization. However, the opposite condition of phage pre-existence in the GOACs did not lead to more encouraging outcomes, either; the phage pre-treatment of the GOACs proved to be unable to prevent ulterior bacterial colonization of the GOACs as opposed to previous findings from comparable assays [52].

Our *E. coli* GOAC assays generated results that seem contradictory to some of Green and colleagues’ findings after conducting similar assays in mice. Indeed, as opposed to their results, MBPs of phage ES17 could not revert the bacteria-favoring scenario of our *E. coli*-infected GOACs compared to phage HP3, which is devoid of MBPs. ES17 did not perform better at lowering GOAC bacterial titers than HP3 in any but one culture condition. Indeed, a slightly better effect at 24 h was observed during continuous phage infusion condition, achieving significantly lower bacterial titer levels than the bacteria-only control GOACs but not significantly lower than the HP3 GOACs. This is highly contrasting with the spectacular bactericidal efficiency of ES17 in Green and colleagues’ mice model, especially compared to the inefficiency of HP3. Several factors might explain these differences. First, the GOACs used in this work do not replicate the complex aero-anaerobic gradients found in the live digestive tract, though previous complex iterations of GOACs have been able to achieve this [22]. For example, as a facultative aero-anaerobe, *E. coli* has been shown to endure dynamic gene regulation during gut colonization to handle the aero-anaerobic switch in parallel to the development of phage-resistant mechanisms when phages were co-introduced in the gut [66]. Anaerobic conditions have been suspected to influence phage–bacteria interactions, including the adsorption rates and frequency of BPR-inducing mutations [73]. Second, it has been increasingly documented that phages, while incapable of actively infecting eukaryotic cells, can become internalized in them. This is notably the case in the gut, where phages in the digestive lumen are subject to an epithelial uptake by transcytosis, representing a potential sink for phages and limiting their in-gut availability [74]. It is unknown whether the intensity of this uptake-mediated loss in available phages is similar in GOACs and mice. Third, in relation to the aforementioned digestive phage transcytosis mechanisms, GOACs lack any immune system. While increasing evidence is advocating significant interactions between gut-resident phages and the mucosal immune system, notably explained via their transcytosis potential in both health and disease, little is known about the acute implication of the immune system on newly introduced phages in the gut community in the scenario of oral phage therapy [75,76]. The idea that phage–immune interactions could result in increased bactericidal properties is supported by previous findings [77]. Fourth, the tissue architecture in GOACs probably only resumes part of the complexity of that of the live digestive tract: the aforementioned spatial determinants influencing gut-specific phage–bacteria coexistence are thus likely different, too [67]. Fifth, Green and colleagues’ MBP-dependent bactericidal effect is mostly reported in mice’s colons: this specific intestinal segment might not be accurately modeled by the use of Caco-2 cells which, despite their colic origin, tend to reconstitute a phenotype closer to that of the small intestine, though the addition of LS 174T cells might mitigate this mismatch [78]. Lastly, and perhaps most importantly, the GOACs used in this work lack any sort of intra- and inter-specific bacterial competition, as the only bacterial strain present in a GOAC model is the one directly targeted by the co-introduced phages. This is a critical limitation, as these GOACs will likely not reproduce the synergistic bactericidal action mediated by phage therapy’s lytic pressure and the relative fitness costs associated with the development of bacterial “escape” growth under this pressure in a competitive microbiological environment. This study thus fails to reproduce one example in the larger family of “vice effects” that are highly suspected to influence phage therapy’s outcomes [13]. In line with this hypothesis, the recent findings of Forsyth and colleagues have illustrated that even though the fitness costs associated with BPR itself in digestive *E. coli* ST131 were not intrinsically high, a significant synergistic bactericidal effect against *E. coli* ST131 could be achieved by combining phage therapy with a probiotic *E. coli* Nissle, leading to a dramatic increase in BPR-associated fitness costs [79]. It should also be noted that, either in Green and colleagues’ work or in ours, comparing ES17 with HP3 does not allow for a proper evaluation of the net, with isolated properties of ES17’s MBPs. This could only be achieved by comparing wild-type ES17 with an engineered loss-of-function mutant of this phage, similarly to what others have investigated with anti-*E. coli* phage T4 and its MBP-inducing outer capsid protein Hoc [52].

Besides the aforementioned factors potentially explaining GOAC–mice mismatch, there are a number of other limitations to our work. GOACs are work-intensive models. Developing and validating them in a reproductible fashion starting from no previous experience will often require a lot of troubleshooting. Recent ambitious efforts have been made in the development of “next-generation organ-on-chip” through the use of complex models, including both “in parallel” (i.e., double-channel GOACs) and/or “in series” combined models, such as a “gut-liver-axis-on-a-chip” [22,80]. These rightfully generate much enthusiasm for the future of complex animal-free experimental models. Yet, while a user can vastly increase the model’s complexity and physiological relevance by implementing a baseline simplified healthy gut microbiota and working with double-channel GOACs, anaerobic culture chambers, peristaltic pumps, oxygen sensors, heat-inactivating chips, self-dispensing output pumps, or other custom features, all of these additional aspects will increase the tedious nature of GOAC development and handling [22,24]. This limitation also restricts numerical reproducibility: a single user working with a single multichannel pump can only handle series of a certain number of GOACs at a time, and the delay needed to bring them from liquid PDMS to ready-to-infect GOACs creates a certain time inertia. For this reason, our will to test numerous conditions in a limited timeframe led us to work only with triplicates, which sometimes showed suboptimal internal reproducibility. This was, for example, the case with simultaneous phage–bacteria introduction conditions, which were already shown to generate considerable stochastic variations, with each GOAC model representing a unique, idiosyncratic ecosystem [24]. Another limitation to this work is the main focus on bacterial outcomes at the expense of phage outcomes. Phages were not quantified in egressing a GOAC medium to establish the in-GOAC evolution of the PFU/mL phage titers in parallel to bacterial titers; likewise, co-evolved phages were not retrieved for a comparative genomic analysis. An example of such parallel monitoring has been reported using quantitative polymerase chain reaction (qPCR) in a way that can be automated [24]. Such automation can also allow for significantly finer time granularity compared to our once-a-day monitoring method. On the other hand, plating-based CFU monitoring allowed us to screen, isolate, and type phage- and/or GOAC-induced bacterial variants in a way that qPCR could not have achieved if used alone. These approaches might be seen as complementary but could also be complemented or, in some cases, replaced by shotgun metagenomics.

In conclusion, we investigated bacterial dynamics under phage pressure in GOACs, first with *P. aeruginosa* and corresponding phage PNM, and then with *E. coli* and corresponding phages ES17 and HP3. These assays were modulated around numerous conditions of phage titers, phage MBPs, phage–bacteria timing of introduction, and epithelial cell lines. GOACs were shown to achieve relevant mucin secretion with an optimized secretion of MUC2 in bicellular GOACs, which were mostly used in this work. Though the aforementioned varied growth conditions generated distinct results, our assays have drawn a general trend of bacterial prosperity and resilience in these models, showing a significantly higher rate of “escape” growth under phage lytic pressure than in more neutral and static in vitro assays. Comparative assays illustrated that the serum-induced loss of phage activity might explain this mismatch for *P. aeruginosa*, but likely not for *E. coli*, at least considering the three phages we worked with. Other factors increasing bacterial advantage were the use of bicellular GOACs and the temporal pre-existence of the bacteria in the model compared to phages. In *E. coli* assays, the temporal pre-existence of phages or continuous infusion of phages in the model could not shift this balance of power. Increasing the MOI of single phages or using a two-phage combination led to temporarily lower bacterial titer levels, though they never reached the threshold of bacterial undetectability at any timepoint. The phenotypic and genomic characterization of bacterial variants thriving under phage pressure suggests that achieving phage evasion can either be linked to durable genomic modifications causing BPR or to suspected dynamic gene regulation and non-bacterial phage evasion determinants. The contributions of these respective mechanisms might greatly vary between bacterial species, as might the serum-related loss of phage activity. The occurrence of this suspected dynamic gene regulation was not directly confirmed in our model, highlighting the need for transcriptomic or proteomic monitoring in future GOAC-mediated phage research, for example. Importantly, in *E. coli* assays, this gut-specific bacterial prosperity could not be reverted by the use of a phage harboring MBPs (ES17) compared to an MBP-lacking phage (HP3), potentially contradicting the recent findings from similar assays in mice models. The numerous differences between GOACs and mice models might explain these differences. Various perspectives pave the way for future phage research in GOACs or other digestive models, like the critical need for intra- and inter-species bacterial competition, the investigation of a “block and replace” synergistic therapy using a phage–probiotic combination, the study of BPR- and non-BPR-associated fitness costs, the extrapolation of phage–antibiotic interactions in these models, and the comparative genomic analysis between phage-escaping bacterial variants from GOACs compared to those from control static under in vitro conditions.

## Figures and Tables

**Table 1:** Typing of *P. aeruginosa* variants. Summary of phage susceptibility testing and genomic characterization of 9 variants of *P. aeruginosa* CN573 retrieved after co-evolution with phage PNM in GOACs.

Isolate	Colony Phenotype	PNM EOP	Phage PNM Susceptibility	Mutations in Genes	Structural Variation
CN573-V1	Bright green big colonies	0.0009	Partly resistant	Frameshift variant in *pilM*, stop gained in *exsC*, missense variant in *eutG*	
CN573-V2	Bright green big colonies	0.0010	Partly resistant	Inframe deletion in *pilT*, stop gained in *exsC*	
CN573-V3B	Brown big colonies	0	Fully resistant	Frameshift variant in *pilQ*, stop gained in *exsC*, missense variant in *fabG*	Chromosomal deletion (including *galU* and *hmgA*)
CN573-V3S	Brown small colonies	0	Fully resistant	Frameshift variant in *pilQ*, stop gained in *exsC*, missense variant in *fabG*	Chromosomal deletion (including *galU* and *hmgA*)
CN573-V4	Brown small colonies	0	Fully resistant	Frameshift variant in *pilP*, stop gained in *exsC*	Chromosomal deletion (including *galU* and *hmgA*)
CN573-V5	Bright green big colonies	0	Fully resistant	Frameshift variant in *pilQ*, stop gained in *exsC*, missense variant in *fabG*	Chromosomal deletion
CN573-V6	Pale green small colonies	0	Fully resistant	Frameshift variant in *pilP* and *wapR*, stop gained in *exsC*	
CN573-V7	Brown small colonies	0	Fully resistant	Frameshift variant in *pilC*, stop gained in *exsC*, and missense variant in *fabG*	Chromosomal deletion (including *galU* and *hmgA*)
CN573-V8	Pale green irregular colonies	0	Fully resistant	Stop gained in *exsC* and *pilO*, missense variants in *fabG* and *algC*	

**Table 2:** Typing of *E. coli* variants. Summary of phage susceptibility testing and genomic characterization of 5 variants of *E. coli* ST131 retrieved after co-evolution with phage ES17 or HP3 in GOACs.

Isolate	Colony Phenotype	Inductor Phage	Inductor Phage’s EOP	Inductor Phage’s Susceptibility	Mutations and/or Structural Variations
ST131-V1	Bright white dwarf colonies	HP3	0.116	Partly susceptible	No detectable non-synonymous variation
ST131-V2	Bright white colonies	ES17	1	Fully susceptible	No detectable non-synonymous variation
ST131-V3	Hazy gray large colonies	ES17	1	Fully susceptible	Deletion causing a frameshift variant in a hypothetical protein
ST131-V4	Bright grey colonies	HP3	1	Fully susceptible	No detectable non-synonymous variation
ST131-V5	Bright white colonies	ES17	1	Fully susceptible	No detectable non-synonymous variation

## Data Availability

Sequencing data regarding *P. aeruginosa* variants has been uploaded to SRA with the BioProject accession number PRJNA1039750 and can be accessed at the following link: https://dataview.ncbi.nlm.nih.gov/object/PRJNA1039750?reviewer=jpoe94tkdc37jf884ov8odjt74 (accessed on 24 April 2024). Sequencing data regarding the *E. coli* variants can be similarly accessible through the BioProject accession number PRJNA1041074.

## Supplementary Materials

The following supporting information can be downloaded at: https://www.mdpi.com/article/10.3390/v16071047/s1. Table S1: disk-diffusion antibiotic susceptibility testing results of reference bacterial strains used in this work, *P. aeruginosa* CN573 and *E. coli* ST131.
